# Contribution of tetrodotoxin-resistant persistent Na^+^ currents to the excitability of C-type dural afferent neurons in rats

**DOI:** 10.1186/s10194-022-01443-7

**Published:** 2022-06-28

**Authors:** Michiko Nakamura, Il-Sung Jang

**Affiliations:** 1 Department of Pharmacology, School of Dentistry, Kyungpook National University, Daegu, 700-412 Republic of Korea; 2 Brain Science & Engineering Institute, Kyungpook National University, Daegu, 700-412 Republic of Korea

**Keywords:** Migraine, Dural afferent neurons, TTX-R Na^+^ channels, Persistent Na^+^ currents, Patch-clamp

## Abstract

**Background:**

Growing evidence supports the important role of persistent sodium currents (I_NaP_) in the neuronal excitability of various central neurons. However, the role of tetrodotoxin-resistant (TTX-R) Na^+^ channel-mediated I_NaP_ in the neuronal excitability of nociceptive neurons remains poorly understood.

**Methods:**

We investigated the functional role of TTX-R I_NaP_ in the excitability of C-type nociceptive dural afferent neurons, which was identified using a fluorescent dye, 1,1′-dioctadecyl-3,3,3′,3′-tetramethylindocarbocyanine perchloride (DiI), and a whole-cell patch-clamp technique.

**Results:**

TTX-R I_NaP_ were found in most DiI-positive neurons, but their density was proportional to neuronal size. Although the voltage dependence of TTX-R Na^+^ channels did not differ among DiI-positive neurons, the extent of the onset of slow inactivation, recovery from inactivation, and use-dependent inhibition of these channels was highly correlated with neuronal size and, to a great extent, the density of TTX-R I_NaP_. In the presence of TTX, treatment with a specific I_NaP_ inhibitor, riluzole, substantially decreased the number of action potentials generated by depolarizing current injection, suggesting that TTX-R I_NaP_ are related to the excitability of dural afferent neurons. In animals treated chronically with inflammatory mediators, the density of TTX-R I_NaP_ was significantly increased, and it was difficult to inactivate TTX-R Na^+^ channels.

**Conclusions:**

TTX-R I_NaP_ apparently contributes to the differential properties of TTX-R Na^+^ channels and neuronal excitability. Consequently, the selective modulation of TTX-R I_NaP_ could be, at least in part, a new approach for the treatment of migraine headaches.

**Supplementary Information:**

The online version contains supplementary material available at 10.1186/s10194-022-01443-7.

## Background

Sensory neurons of the dorsal root ganglia (DRG) and trigeminal ganglia (TG) express multiple types of voltage-gated Na^+^ channels, which are mainly classified into tetrodotoxin-sensitive (TTX-S; e.g., Na_V_1.1, Na_V_1.2, Na_V_1.6, and Na_V_1.7) and TTX-resistant (TTX-R; e.g., Na_V_1.8 and Na_V_1.9) subtypes [1]. The study of Na_V_1.8 has shed light on nociceptive transmission because it is activated and inactivated at depolarized potentials and involved in the generation and conduction of action potentials in response to sustained depolarizing stimuli [1–4]. Moreover, Na_V_1.8 is closely related to the development and maintenance of inflammatory hyperalgesia as it is subjected to peripheral sensitization by inflammatory mediators (IMs) such as prostaglandin E_2_ (PGE_2_) [5, 6]. In studies on Na_V_1.8, the subtype has been found to mediate different properties of TTX-R Na^+^ currents (I_Na_). For example, the extent of use-dependent inhibition, which can be pivotal to the repetitive generation of action potentials at depolarized membrane potentials, has varied among studies involving sensory neurons [7–10]. Although differences in the experimental conditions, such as the solutions, holding potentials, species, ages of animals, and neuronal populations used, potentially explain the observed discrepancies between previous studies, the mechanisms underlying the diverse properties of TTX-R Na^+^ channels expressed in sensory neurons remain largely unknown.

In general, voltage-gated Na^+^ channels are rapidly inactivated after their full activation. However, some noninactivating currents, also known as persistent sodium currents (I_NaP_), are known to exist [11]. Growing evidence indicates that I_NaP_ contribute to the firing patterns of neurons, such as tonic and sustained firing, under normal physiological conditions in various brain regions [12–14], and an abnormal increase in I_NaP_ has been associated with several neurological disorders, including epilepsy and pain [15–17]. In studies of nociceptive neurons, Na_V_1.9 has been identified as a subtype responsible for TTX-R I_NaP_ based on its slow kinetics [18.19], and this subtype may be involved in the regulation of subthreshold neuronal excitability [19, 20]. A previous study of human Na_V_1.8 found that it produces a larger persistent current than that shown in rat Na_V_1.8, and these Na_V_1.8 current properties were related to increased firing frequency in neurons [21]. However, the role of Na_V_1.8-mediated I_NaP_ in the excitability of nociceptive neurons remains poorly understood. Therefore, in the present study, we tested the hypothesis that Na_V_1.8-mediated I_NaP_ are related to channel properties, and consequently, affect the excitability of C-type dural afferent neurons, which we identified using a retrograde fluorescent dye, 1,1′-dioctadecyl-3,3,3′,3′-tetramethylindocarbocyanine perchloride (DiI).

## Materials and methods

All experiments were conducted in accordance with approved animal protocols and guidelines established by the Animal Care Committee of Kyungpook National University (Approval No. KNU-2017–0052). The animal studies are reported in compliance with the ARRIVE guidelines [22], and every effort was made to minimize the number of animals used and their suffering.

### Preparation

Neurons within the TG innervating the dura were identified after the application of the retrograde tracer DiI to the dura, as previously described [23, 24]. Briefly, male Sprague Dawley rats (three to four weeks old; Samtako, Osan, Republic of Korea) were intraperitoneally anesthetized with a mixture of ketamine (20 mg/kg) and xylazine (10 mg/kg). The cranial bone overlying the superior sagittal sinus was gently removed using a careful craniotomy procedure involving a dental drill, and the dura was exposed with a burr hole of 2 mm. Ten microliters of DiI solution, in which 100 mg/ml of DiI in dimethyl sulfoxide (DMSO) was diluted 1:10 (v/v) with saline, was applied to the dura. One minute after the application of DiI solution, a dental resin was placed on the exposed dura to replace the removed cranial bone. The incision was closed with sutures, and the rats received intramuscular injections of penicillin G (100,000 U/kg) and naproxen (10 mg/kg) to reduce the risk of postoperative infection and pain. At 7–10 days after the DiI application, the rats were decapitated under ketamine anesthesia (100 mg/kg administered intraperitoneally). A pair of TG were dissected and treated with an external solution [in mM: 150 NaCl, 3 KCl, 2 CaCl_2_, 1 MgCl_2_, 10 glucose and, 10 Hepes (pH 7.4 with Tris-base)] containing 0.3% collagenase (type I) and 0.3% trypsin (type I) for 40–60 min at 37 °C. Thereafter, the TG neurons were dissociated mechanically using trituration with fire-polished Pasteur pipettes in a culture dish (Primaria 3801; Becton Dickinson, Rutherford, NJ, USA). The isolated neurons were used for electrophysiological recordings 2–6 h after preparation. Images of the TG neurons were obtained using a digital microscope camera (ProgRes® MF; Jenoptik, Jena, Germany). For the other subset experiments, TG neurons were obtained from adult male or female rats (8–9 weeks old; Samtako).

### Elvax implantation

Previous studies have shown that acute bath application of a mixture of IMs (1 μM PGE_2_, 10 μM bradykinin, and 1 μM histamine) sensitizes dural afferent neurons and increases their excitability [23, 25, 26]. In addition, bath application of a mixture of IMs increased the peak amplitude of TTX-R I_Na_ [26]. However, we examined whether peripheral sensitization by the application of IMs to the dura mater affected TTX-R I_Na_, including transient and persistent currents and the excitability of dural afferent neurons. Since the identification of dural afferent neurons by the retrograde fluorescent dye DiI takes over 7 days, we applied a mixture of IMs to dural afferent neurons with chronic treatment methods using Elvax (ethylene–vinyl acetate copolymer resin) implantation incorporated with IMs. Although chronic treatment with IMs using Elvax implantation to the dura mater has not been described, Elvax implantation has been widely used for sustained release of small molecules, such as TTX [27], and peptides or proteins [28, 29].

To prepare Elvax implants, beads (100 mg) of Elvax (40 W; DuPont; kindly gifted from JS POLYMER, Seongnam, Republic of Korea) were dissolved in dichloromethane (100 mg/ml) and homogeneously mixed with 20 μl of DMSO containing IMs, i.e., 10 mM PGE_2_, 20 mM bradykinin, and 10 mM histamine. Thereafter, the Elvax solution was dropped on a glass dish using a 1-ml syringe, rapidly frozen, kept at -80 °C for 1 h, and maintained at -20 °C overnight to allow the dichloromethane to evaporate. To implant an Elvax piece containing IMs, male Sprague Dawley rats (3–4 weeks old; Samtako) were intraperitoneally anesthetized with a mixture of ketamine (20 mg/kg) and xylazine (10 mg/kg). Two burr holes (2 mm diameter) were made using a careful craniotomy procedure, and the dura was exposed. DiI solution and a small Elvax piece (approximately 2 × 2 × 2 mm, 8.0 ± 0.3 mg, *n* = 11) were placed on the dura mater through each hole in the cranial bone, and a dental resin was placed on the exposed dura to replace the removed cranial bone. The Elvax piece was usually left on the dura mater from the day of implantation until the day of electrophysiological recording (7–10 days). The implantation of Elvax containing vehicle (DMSO) or IMs did not cause deficits in the general motor behaviors of rats. Considering that IMs were equally distributed in Elvax, the amounts of PGE_2_, bradykinin, and histamine in the implanted Elvax piece (8 mg) were approximately calculated to be 2.9, 340, and 5.6 μg, respectively. In the present study, we did not measure the total estimated concentration of IMs in the dural tissue from the Elvax piece, the duration of IM release after the implantation of Elvax, and the residual amounts of IMs within the implanted Elvax piece after the animals were euthanized. However, biomolecules or chemicals (< 2 kDa), such as basic fibroblast growth factor, MK-801, and doxycycline, embedded in the Elvax, have been known to be released over 7 days with a peak on the first day [30–32]. In the present study, since we did not perform any behavioral test to examine migraine-like pain behaviors for the Elvax-IM implantation in vivo, we could not verify whether the Elvax-IM implantation in the dura mater induces migraine-like pain behaviors. Further behavioral studies are needed to validate the Elvax-IM implantation as a chronic inflammation model for peripheral sensitization of the dura mater.

### Electrical measurements

All electrical measurements were performed using conventional whole-cell patch recordings and a standard patch-clamp amplifier (Axopatch 200B; Molecular Devices, Union City, CA, USA). Neurons were voltage-clamped at a holding potential (V_H_) of -80 mV, except where indicated. Patch pipettes were produced from borosilicate capillary glass (G-1.5; Narishige, Tokyo, Japan) using a pipette puller (P-97; Sutter Instrument Co., Novato, CA, USA). The resistance of the recording pipettes filled with the internal solution was 0.7–1.2 MΩ. Membrane potentials were corrected for the liquid junction potential, and the pipette capacitance and series resistance (60%–90%) were compensated for. DiI-positive TG neurons were viewed under phase contrast or fluorescence using an inverted microscope (TE2000; Nikon, Tokyo, Japan). Membrane currents were filtered at 3–5 kHz, digitized at 10–20 kHz, and stored on a computer equipped with pCLAMP 10.7 (Molecular Devices). Capacitative and leak currents were subtracted using the P/4 subtraction protocol of pCLAMP 10.7. To record the TTX-R I_Na_, the internal solution was composed of the following (in mM): 140 CsF, 10 CsCl, 2 EGTA, 2 Na_2_-ATP, and 10 Hepes (pH 7.2 with Tris-base). In the current-clamp experiments, both CsF and CsCl were replaced with equimolar KF and KCl, respectively. All experiments were performed at room temperature (22 ºC–25 ºC). The bath solution used was composed of the following (in mm): 130 NaCl, 20 TEA-Cl, 2 CaCl_2_, 1 MgCl_2_, 10 Hepes, 10 glucose, 0.01 CdCl_2_, and 0.0003 TTX (pH 7.4 with Tris-base).

### Data analysis

The peak amplitude of transient TTX-R I_Na_ was measured by subtracting the baseline from the peak amplitude of TTX-R I_Na_ using pCLAMP 10.7. The amplitude of the steady-state component of transient TTX-R I_Na_ was measured by subtracting the baseline from the mean amplitude of TTX-R I_Na_ at 90–95 ms. For experiments in which the voltage-activation relationship was assessed, the extracellular Na^+^ concentration was reduced to 20 mM by replacing 110 mM NaCl with equimolar *N*-methyl-D-glucamine-Cl, and the amplitude of TTX-R I_Na_ was transformed into conductance (G) using the following equation:

G = I / (V—E_Na_),

where E_Na_ is the Na^+^ equilibrium potential calculated using the Nernst equation. The voltage-activation and voltage-inactivation relationships of TTX-R Na^+^ channels were fitted to Boltzmann equations, respectively, as follows:

G/G_max_ = 1/{1 + exp[(V_50,activation_—V)/*k*]} and I/I_max_ = 1—1/{1 + exp[(V_50,inactivation_—V)/*k*]},

where G_max_ and I_max_ are the maximum conductance and current amplitude, respectively, V_50,activation_ and V_50,inactivation_ are half-maximal voltages for activation and fast inactivation, respectively, and *k* is the slope factor. The kinetic data for the recovery from inactivation were best fitted to the triple exponential function using the following equation:

I(*t*) = A_0_ + A_fast_ × [1—exp(-*t*/τ_fast_)] + A_intermediate_ × [1—exp(-*t*/τ_intermediate_)] + A_slow_ × [1—exp(-*t*/τ_slow_)],

where I(*t*) is the amplitude of TTX-R I_Na_ at time *t*, and A_fast_, A_intermediate_, and A_slow_ are the amplitude fraction of τ_fast_, τ_intermediate_, and τ_slow_, respectively. The kinetic data for the development of inactivation were best fitted to the double exponential function using the following equation:

I(*t*) = A_0_ + A_fast_ × [exp(-*t*/τ_fast_)] + A_slow_ × [exp(-*t*/τ_slow_)],

where I(*t*) is the amplitude of TTX-R I_Na_ at time *t*, and A_fast_ and A_slow_ are the amplitude fraction of _fast_ and _slow_, respectively. Numerical values are provided as the mean ± standard error of the mean (SEM) using values normalized to the control. Significant differences were tested using unpaired t-tests, except where indicated. P values of < 0.05 were considered statistically significant. In the current-clamp experiments, the rheobase currents, which represent the minimal currents that generate action potentials, were determined using a square depolarizing current injection in 10 pA increments (500 ms duration).

### Drugs

The following drugs were used in this study: riluzole, collagenase, trypsin, TTX, DiI, prostaglandin E_2_, bradykinin, histamine (all from Sigma, St. Louis, MO, USA), A803467, and ZD7288 (the latter two from Tocris, Bristol, England). ZD7288 and TTX were dissolved in distilled water to give a stock solution of 50 mM and 3 mM, respectively. Riluzole and A803467 were dissolved in DMSO to give a stock solution of 100 mM and 1 mM, respectively. The final concentration of DMSO applied to the external solution was ≤ 0.1% v/v, and DMSO (0.1% v/v) did not affect the TTX-R I_Na_ (Supplementary Fig. S1A). Extracellular solutions containing the drugs, except collagenase, trypsin, and DiI, were applied using the “Y-tube system” for rapid solution exchange [33].

### Results

## Persistent Na^+^ currents mediated by TTX-R Na^+^ channels in dural afferent neurons

**Fig. 1:**
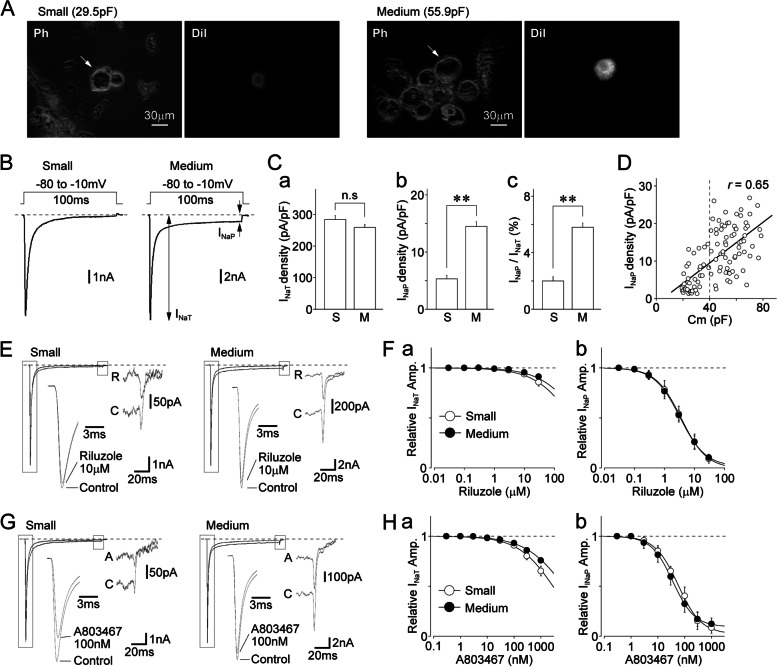
Persistent sodium currents mediated by TTX-R Na^+^ channels in dural afferent neurons.** A.** Typical phase contrast (Ph) and fluorescence (DiI) images of small- (left) and medium-sized (right) DiI-positive neurons. **B.** Typical traces of TTX-R I_Na_ recorded in small- and medium-sized DiI-positive neurons. TTX-R I_Na_ was elicited by voltage step pulses (100 ms duration; -10 mV depolarization at a V_H_ of -80 mV). The noninactivating persistent I_Na_ (I_NaP_) was followed by transient I_Na_ (I_NaT_) in DiI-positive neurons. **C.** The mean values of the density of TTX-R I_NaT_ (**a**) and I_NaP_ (**b**) and the amplitude ratio of I_NaP_/I_NaT_ (**c**) in small- (S) and medium-sized (M) DiI-positive neurons. Columns and error bars represent the mean and SEM from 31 small- and 40 medium-sized DiI-positive neurons. **; *p* < 0.01, n.s; not significant (unpaired t-test). **D.** Scatter plot of the density of TTX-R I_NaP_ against membrane capacitance (Cm) obtained from the same neurons (*n* = 71). The linear trend line represents the best fit using a least-squares fit (*r* = 0.65). **E.** Typical traces of TTX-R I_Na_ recorded in the absence and presence of 10 μM riluzole in small- (left) and medium-sized (right) DiI-positive neurons. Inset traces show TTX-R I_NaT_ and I_NaP_ with expanded time and amplitude scales, respectively. **F.** Concentration–response relationships of riluzole for TTX-R I_NaT_ (**a**) and I_NaP_ (**b**). The points and error bars represent the mean and SEM from seven small- and seven medium-sized DiI-positive neurons. **G.** Typical traces of TTX-R I_Na_ recorded in the absence and presence of 100 nM A803467 in small- (left) and medium-sized (right) DiI-positive neurons. Inset traces show TTX-R I_NaT_ and I_NaP_ with expanded time and amplitude scales, respectively. **H.** Concentration–response relationships of A803467 for TTX-R I_NaT_ (**a**) and I_NaP_ (**b**). The points and error bars represent the mean and SEM from seven small- and seven medium-sized DiI-positive neurons

In our previous study, based on the expression of calcitonin gene-related peptide (CGRP) and transient receptor potential vanilloid 1, we showed that most small- and medium-sized dural afferent neurons are nociceptive C-type neurons [24]. These neurons express TTX-R Na^+^ channels, but they differ considerably in terms of firing patterns in response to depolarizing current stimuli [24]. To investigate the functional role played by TTX-R Na^+^ channels in the excitability of these neurons, we first examined the properties of TTX-R I_Na_ in small- and medium-sized DiI-positive neurons, which were identified using the fluorescent dye DiI (Fig. 1A). The neuronal size of DiI-positive neurons was classified according to two parameters: diameter (the means of the shortest and longest axes: < 30 μm for small-sized and 30–40 μm for medium-sized) and membrane capacitance (< 40 pF for small-sized and 40–80 pF for medium-sized). Electrophysiological data from DiI-positive neurons that showed discordance in these two parameters were discarded.

TTX-R I_Na_ were elicited by depolarizing voltage steps to -10 mV at a V_H_ of -80 mV using a CsF-based pipette solution in the presence of both 300 nM TTX and 100 μM Cd^2+^ in C-type DiI-positive neurons (Fig. 1B). To minimize the effect of Na_V_1.9 currents, all electrophysiological data were obtained at least 5–10 min after making the whole-cell configuration. The current density of the transient TTX-R I_Na_ (I_NaT_) was slightly larger in small-sized DiI-positive neurons than that in the medium-sized versions [284.0 ± 12.6 pA/pF (*n* = 39) vs. 259.0 ± 9.2 pA/pF (*n* = 66), respectively; *p* < 0.01; Fig. 1Ca]. However, the noninactivating or persistent component of TTX-R I_Na_ (i.e., I_NaP_) was observed in most medium-sized DiI-positive neurons (Fig. 1B). The density of TTX-R I_NaP_ was larger in medium-sized DiI-positive neurons than that in the small-sized versions [14.5 ± 0.8 pA/pF (*n* = 66) vs. 5.3 ± 0.6 pA/pF (*n* = 39), respectively; *p* < 0.01; Fig. 1Cb]. In addition, the amplitude ratio for I_NaT_ and I_NaP_ (I_NaP_/I_NaT_) was larger in medium-sized DiI-positive neurons than that in the small-sized ones [5.8% ± 0.3% (*n* = 66) vs. 2.0% ± 0.3% (*n* = 39), respectively; *p* < 0.01; Fig. 1Cc]. The density of TTX-R I_NaP_ was correlated with neuronal capacitance (*r* = 0.65, *n* = 105, *p* < 0.01; Fig. 1D). In addition, in both small- and medium-sized DiI-positive neurons, TTX-R I_NaP_ were potently inhibited by riluzole, a specific persistent sodium current inhibitor [34, 35] [IC_50_ values: 3.6 ± 0.2 μM (*n* = 7) and 3.3 ± 0.3 μM (*n* = 7), respectively; Fig. 1E, F], and by A803467, a selective Na_V_1.8 blocker [IC_50_ values: 57.3 ± 9.8 nM (*n* = 7) and 36.2 ± 6.2 nM (*n* = 7), respectively; Fig. 1G, H].

**Fig. 2:**
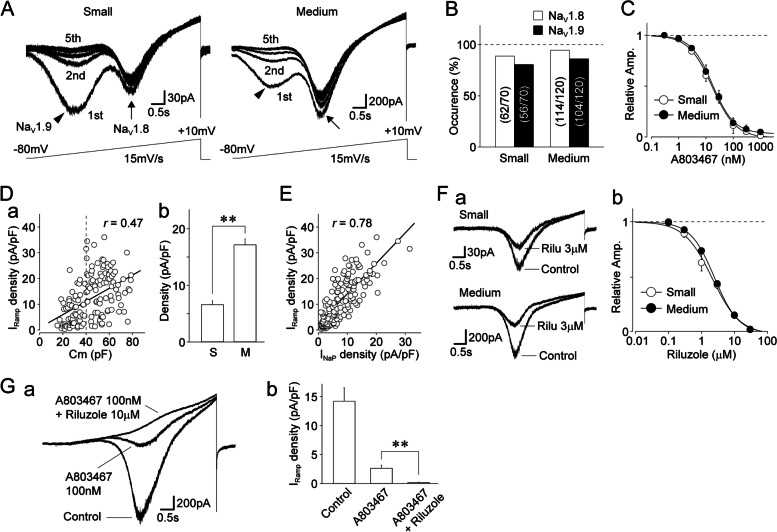
Slow voltage-ramp-induced currents mediated by TTX-R Na^+^ channels in dural afferent neurons.** A.** Typical traces of slow voltage-ramp-induced TTX-R I_Na_ recorded in small- (**a**) and medium-sized (**b**) DiI-positive neurons. TTX-R I_Na_ were elicited by five successive voltage-ramp stimuli every 15 s (6 s duration; up to + 10 mV depolarization at a V_H_ of -80 mV; 15 mV/s). Notably, voltage-ramp stimuli elicited “W-shaped” membrane currents, which are potentially mediated by Na_V_1.9 (left, arrowheads) and Na_V_1.8 (right, arrows). Moreover, the Na_V_1.9-mediated ramp currents rapidly and completely disappeared within 3 min. **B.** Occurrence of Na_V_1.8- and Na_V_1.9-mediated ramp currents in small- and medium-sized DiI-positive neurons. The values in parenthesis represent the number of DiI-positive neurons showing Na_V_1.8- and Na_V_1.9-mediated TTX-R I_Ramp_. The occurrence of Na_V_1.8- and Na_V_1.9-mediated ramp currents was determined immediately after the whole-cell configuration was made. **C.** Concentration–response relationships of A803467 for TTX-R I_Ramp_. The points and error bars represent the mean and SEM from eight small- and seven medium-sized DiI-positive neurons. **D. a**, Scatter plot of the density of TTX-R I_Ramp_ against membrane capacitance (Cm) obtained from the same neurons (*n* = 152). The linear trend line represents the best fit using a least-squares fit (*r* = 0.47). **b**, The mean values of the density of TTX-R I_Ramp_ in small- (S) and medium-sized (M) DiI-positive neurons. The columns and error bars represent the mean and SEM from 60 small- and 92 medium-sized DiI-positive neurons. **; *p* < 0.01 (unpaired t-test). **E.** Scatter plot of the density of TTX-R I_Ramp_ against TTX-R I_NaP_ obtained from the same neurons (*n* = 152). The linear trend line represents the best fit using a least-squares fit (*r* = 0.78). **F. a**, Typical traces of slow voltage-ramp-induced TTX-R I_Na_ recorded in the absence and presence of 3 μM riluzole in small- (upper) and medium-sized (lower) DiI-positive neurons. **b**, Concentration–response relationships of riluzole for the TTX-R I_Ramp_. The points and error bars represent the mean and SEM from six small- and eight medium-sized DiI-positive neurons. **G. a**, Typical traces of slow voltage-ramp-induced TTX-R I_Na_ recorded in the absence and presence of 100 nM A803467 and 100 nM A803467 plus 10 μM riluzole in medium-sized DiI-positive neurons. **b**, Changes in the current density of slow voltage-ramp-induced TTX-R I_Na_ by 100 nM A803467 and 100 nM A803467 plus 10 μM riluzole. The columns and error bars represent the mean and SEM from eight medium-sized DiI-positive neurons. **; *p* < 0.01 (paired t-test)

Persistent sodium currents can be elicited by slow voltage-ramp stimulation [36, 37]. Immediately after making a whole-cell configuration, slow voltage-ramp stimulation elicited “W-shaped” inward currents at a holding potential of -80 mV (Fig. 2A). These ramp currents, which are potentially mediated by Na_V_1.8 and Na_V_1.9, were found in most small- and medium-sized DiI-positive neurons (Fig. 2B). The ramp currents mediated by Na_V_1.9, which were activated at potentials that were more hyperpolarized, completely disappeared within 5 min of making the whole-cell configuration (Fig. 2A). We further analyzed the properties of Na_V_1.8-mediated slow ramp currents after the complete rundown of the left part of these currents. Five minutes after making the whole-cell configuration, the remaining ramp currents had completely disappeared following the addition of a Na^+^-free external solution (data not shown), and they were potently inhibited by A803467 in small- and medium-sized DiI-positive neurons [IC_50_ values: 16.8 ± 2.3 nM (*n* = 8) and 17.3 ± 2.1 nM (*n* = 7), respectively; Fig. 2C]. This confirmed that the right part of the “W-shaped” currents was mediated by Na_V_1.8. The density of Na_V_1.8-mediated ramp currents (I_Ramp_) was correlated with neuronal capacitance (*r* = 0.43, *n* = 152, *p* < 0.01; Fig. 2Da), and the mean density of I_Ramp_ was larger in medium-sized DiI-positive neurons than that in the small-sized ones (17.2 ± 1.0 pA/pF (*n* = 92) vs. 6.6 ± 0.7 pA/pF (*n* = 60), respectively; *p* < 0.01; Fig. 2Db). The density of Na_V_1.8-mediated I_Ramp_ was also correlated with the density of TTX-R I_NaP_ (*r* = 0.78, *n* = 152, *p* < 0.01; Fig. 2E). Na_V_1.8-mediated I_Ramp_ were significantly inhibited by riluzole in small- and medium-sized DiI-positive neurons [IC_50_: 2.1 ± 0.3 μM (*n* = 6) and 2.5 ± 0.2 μM (*n* = 8), respectively; Fig. 2F]. Na_V_1.8-mediated I_Ramp_ were greatly reduced by 100 nM A803467 (19.6 ± 7.0% of the control, *n* = 8) and the residual currents were completely attenuated by the cumulative application of 100 nM A803467 and 10 μM riluzole (1.4 ± 1.3% of the control, *n* = 8, *p* < 0.01, Fig. 2G).

In the present study, we used 4–5 week old male rats to investigate the role of TTX-R I_NaP_ in the excitability of dural afferent neurons. We used young male rats to minimize the variables mediated by hormones and/or neurosteroids that fluctuate due to the menstrual cycle, such as progesterone, pregnenolone, and allopregnanolone, as the serum levels of these compounds are associated with chronic headache disorders [38, 39]. It has been shown that migraine prevalence peaks in females’ post-puberty [40, 41] and that there are both quantitative and qualitative differences in the IM-induced sensitization of dural afferents [26]. We examined the basal properties of TTX-R I_NaP_ in DiI-positive neurons isolated from adult male and female rats (8–9 weeks old). In these experiments, we found that the density of Na_V_1.8-mediated I_NaP_ or I_Ramp_ was highly correlated to neuronal size in DiI-positive neurons isolated from adult male and female rats (Supplementary Fig. S1B-E). The density of Na_V_1.8-mediated I_NaP_ or I_Ramp_ in small-sized or medium-sized DiI-positive neurons of adult rats was not statistically different from that of young male rats (Supplementary Fig. S1B-E). This finding suggests that the basal properties of TTX-R I_NaP_ in dural afferent neurons do not differ depending on animals’ age and/or sex. However, further studies are needed to reveal whether chronic inflammation induces an increase in the density of TTX-R I_NaP_ in DiI-positive neurons in adult male/female rats.

### Voltage dependence of TTX-R Na^+^ channels in dural afferent neurons

The voltage-activation relationship of TTX-R Na^+^ channels was examined in small- and medium-sized DiI-positive neurons. In these experiments, extracellular Na^+^ concentration was reduced to 30 mM to improve the quality of voltage clamping. TTX-R I_Na_ were recorded using 100 ms depolarizing test pulses from -80 mV to + 20 mV in 10 mV increments (Supplementary Fig. S2A). The conductance data were then normalized to the maximal conductance and fitted to the Boltzmann function (Supplementary Fig. S2A). However, the midpoint voltages for activation (V_50,activation_) did not differ between the two groups of DiI-positive neurons [V_50,activation_: -22.8 ± 0.3 mV *(n* = 32) and -22.3 ± 0.3 mV (*n* = 44) in small- and medium-sized DiI-positive neurons, respectively; *p* = 0.25]. In addition, the V_50,activation_ values were not related to cell capacitance (*r* = 0.01, n = 76, p = 0.95; Supplementary Fig. S2B) or the density of TTX-R I_NaP_ (*r* = 0.02, *n* = 76, *p* = 0.85; Supplementary Fig. S2C). Contrastingly, Na_V_1.9-mediated TTX-R I_Na_ were clearly observed at a V_H_ of -120 mV, where the current density of Na_V_1.9-mediated TTX-R I_Na_ did not differ between the small- and medium-sized DiI-positive neurons (Supplementary Fig. S2D, E).

**Fig. 3:**
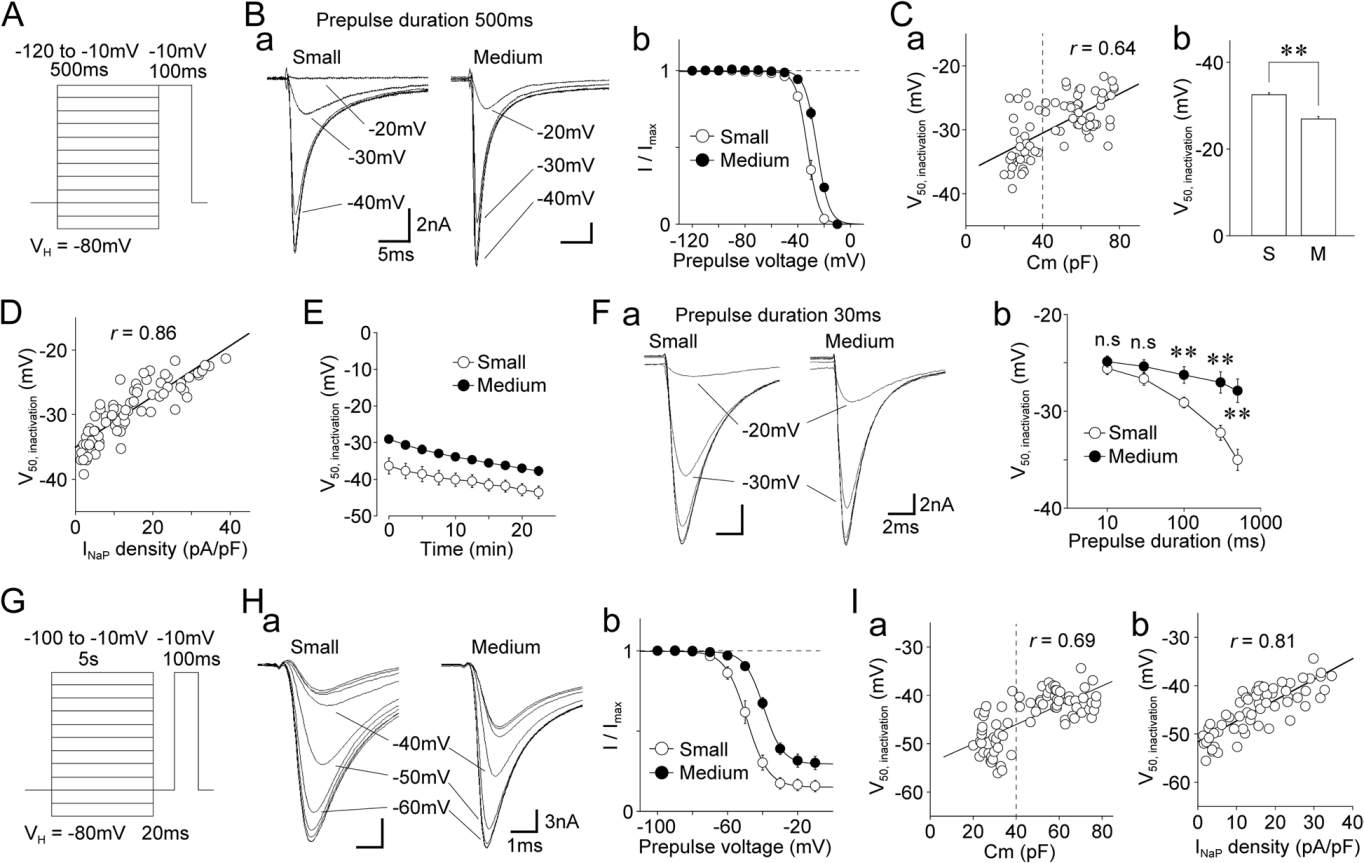
Voltage-inactivation relationships of TTX-R Na^+^ channels in dural afferent neurons. **A.** Schematic illustration of voltage step pulses for the steady-state fast inactivation of TTX-R Na^+^ channels. Conditioning prepulses (500 ms duration; -120 mV to -10 mV in 10 mV increments) were immediately followed by test pulses (100 ms duration; up to -10 mV). **B. a**, Typical traces of TTX-R I_Na_ elicited by step pulses shown in **A** obtained from small- (left) and medium-sized (right) DiI-positive neurons. **b**, Voltage-inactivation relationships of TTX-R Na^+^ channels in small- (open circles) and medium-sized (closed circles) DiI-positive neurons. The points and error bars represent the mean and SEM from 32 small- and 44 medium-sized DiI-positive neurons. The continuous lines represent the best fits using a Boltzmann function. **C. a**, Scatter plot of the half-maximal voltage for inactivation (V_50, inactivation_) against membrane capacitance (Cm) (*n* = 76). The linear trend line represents the best fit using a least-squares fit (*r* = 0.64). **b**, The mean values of the V_50, inactivation_ in small- (S) and medium-sized (M) DiI-positive neurons. The columns and error bars represent the mean and SEM from 32 small- and 44 medium-sized DiI-positive neurons. **; *p* < 0.01 (unpaired t-test). **D.** Scatter plot of the half-maximal voltage for inactivation (V_50, inactivation_) against the density of TTX-R I_NaP_ (*n* = 76). The linear trend line represents the best fit using a least-squares fit (*r* = 0.86). **E.** Time-dependent changes in V_50, inactivation_ immediately after making a membrane rupture in small- (open circles) and medium-sized (closed circles) DiI-positive neurons. The points and error bars represent the mean and SEM from six small- and eight medium-sized DiI-positive neurons. **F. a**, Typical traces of TTX-R I_Na_ elicited by voltage step pulses in small- (left) and medium-sized (right) DiI-positive neurons. TTX-R I_Na_ were elicited by test pulses (100 ms duration; up to -10 mV) immediately after conditioning prepulses (10–500 ms duration; -120 mV to -10 mV in 10-mV increments). **b**, Changes in V_50, inactivation_ with various durations (10–500 ms) of depolarizing prepulses. The points and error bars represent the mean and SEM from six small- and eight medium-sized DiI-positive neurons. **; *p* < 0.01, n.s; not significant (unpaired t-test). **G.** Schematic illustration of voltage step protocols for the slow inactivation of TTX-R Na^+^ channels. The conditioning prepulses (5 s duration, -100 mV to -10 mV, 10 mV increments) were followed by the test pulses (100 ms duration, up to -10 mV) with an interval of 100 ms at a potential of -80 mV. **H. a**, Typical traces of TTX-R I_Na_ elicited by voltage step pulses in small- (left) and medium-sized (right) DiI-positive neurons. **b**, Voltage-slow inactivation relationships of TTX-R Na^+^ channels in small- (open circles) and medium-sized (closed circles) DiI-positive neurons. The points and error bars represent the mean and SEM from 32 small- and 44 medium-sized DiI-positive neurons. The continuous lines represent the best fits using a Boltzmann function. **I.** Scatter plots of the half-maximal voltage for inactivation (V_50, inactivation_) against membrane capacitance (Cm) (**a**, *n* = 76) and the density of TTX-R I_NaP_ (**b**, *n* = 76). The linear trend lines represent the best fits using a least-squares fit (**a**, *r* = 0.69; **b**, *r* = 0.81)

The steady-state fast inactivation of TTX-R Na^+^ channels was also examined in small- and medium-sized DiI-positive neurons. TTX-R I_Na_ were recorded using 100 ms depolarizing test pulses to -10 mV following 500 ms prepulses from -120 to -10 mV in 10 mV increments (Fig. 3A, Ba). The peak currents were then normalized to the maximal current recorded at a prepulse of -120 mV, and the data were fitted to the Boltzmann function (Fig. 3Bb). The midpoint voltages for inactivation (V_50,inactivation_) in small-sized neurons were significantly lower than those in medium-sized neurons, where the V_50,inactivation_ values were correlated with cell capacitance (*r* = 0.64, *p* < 0.01; Fig. 3Ca). The mean V_50,inactivation_ values were -32.6 ± 0.7 mV (*n* = 32) and -26.9 ± 0.4 mV (*n* = 44) in small- and medium-sized DiI-positive neurons, respectively (*p* < 0.01; Fig. 3Cb). The V_50,inactivation_ values were further correlated with the density of TTX-R I_NaP_ (*r* = 0.86, *p* < 0.01; Fig. 3D). We found that the values of V_50,inactivation_ were more negative in small-sized DiI-positive neurons than in their medium-sized counterparts, which may have been due to the faster shift in V_50,inactivation_ during the recording, given that F^−^ is known to elicit a hyperpolarizing shift of V_50,inactivation_ values. However, in contrast to Na_V_1.9, there is evidence that Na_V_1.8 is not modulated by F^−^ [42]. Nevertheless, we found a similar time-dependent negative shift in V_50,inactivation_ values for the two types of DiI-positive neurons (Fig. 3E), indicating that a hyperpolarizing shift in V_50,inactivation_ during recording might not be responsible for the difference in V_50,inactivation_ values observed between small- and medium-sized neurons. Therefore, we further examined the effects of prepulse duration on steady-state fast inactivation. The V_50,inactivation_ values were found to be related to prepulse duration in the two DiI-positive neuron types. The voltage dependence of fast inactivation did not differ under prepulse duration conditions of ≤ 30 ms (Fig. 3F). We also found no difference between the V_50,inactivation_ values obtained from TTX-R I_NaT_ and TTX-R I_NaP_ in medium-sized DiI-positive neurons (Supplementary Fig. S1F, G), indicating that both TTX-R I_NaT_ and TTX-R I_NaP_ are mediated by the same channels.

Our finding that the voltage-fast inactivation relationships for TTX-R Na^+^ channels depend on prepulse duration suggests that slow inactivation might underlie the observed difference in V_50,inactivation_ values between the two DiI-positive neuron types. Therefore, we further examined the voltage dependence of slow inactivation of TTX-R Na^+^ channels in small- and medium-sized DiI-positive neurons. TTX-R I_Na_ were elicited using 100 ms depolarizing test pulses to -10 mV following 5,000 ms prepulses from -100 to -10 mV in 10-mV increments (Fig. 3G, Ha). Peak currents were normalized to the maximal current recorded at a prepulse of -100 mV, and the data were fitted to the Boltzmann function (Fig. 3Hb). The V_50,inactivation_ values for slow inactivation were significantly lower in small-sized neurons than those in medium-sized neurons, where V_50,inactivation_ was -49.2 ± 0.8 mV (*n* = 30) and -41.1 ± 0.4 mV (*n* = 41), respectively (*p* < 0.01). The V_50,inactivation_ values were also correlated with cell capacitance (*r* = 0.69, *n* = 71, *p* < 0.01; Fig. 3Ia) and the density of TTX-R I_NaP_ (*r* = 0.81, *n* = 71, *p* < 0.01; Fig. 3Ib).

### Inactivation kinetics of TTX-R Na^+^ channels in dural afferent neurons

**Fig. 4:**
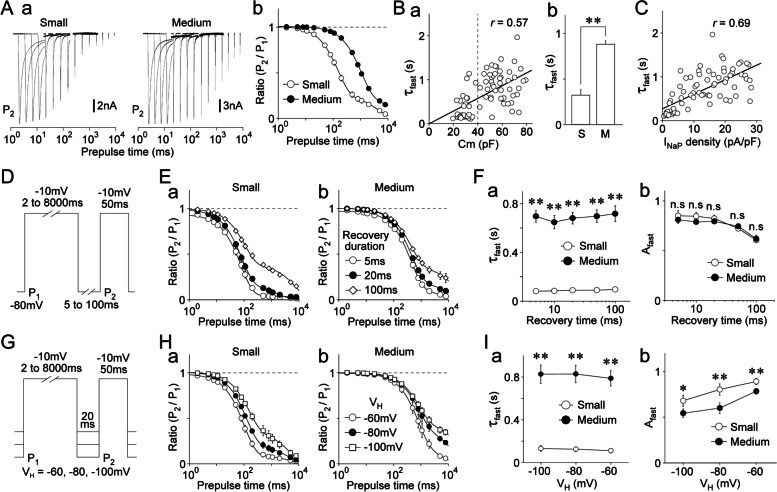
Inactivation kinetics of TTX-R Na^+^ channels in dural afferent neurons.** A. a**, Typical second (P_2_) traces of TTX-R I_Na_ elicited by a two-pulse protocol in small- (left) and medium-sized (right) DiI-positive neurons. TTX-R I_Na_ were induced by the first conditioning pulse (P_1_: -10 mV; 2–8,000 ms duration), which was followed by the second test pulse (P_2_: -10 mV; 50 ms duration) with an interpulse interval of 20 ms at a potential of -80 mV (Supplementary Fig. S2A). **b**, Kinetics for the development of inactivation of TTX-R Na^+^ channels in small- (open circles) and medium-sized (closed circles) DiI-positive neurons. The P_2_/P_1_ ratio of TTX-R I_Na_ was plotted against the duration of the conditioning prepulse. The points and error bars represent the mean and SEM from 28 small- and 47 medium-sized DiI-positive neurons. The continuous lines represent the best fits using a double exponential function. **B. a**, Scatter plot of the weighted time constant (τ_fast_) against membrane capacitance (Cm) (*n* = 75). The linear trend line represents the best fit using a least-squares fit (*r* = 0.57). **b**, The mean values of τ_fast_ in small- (S) and medium-sized (M) DiI-positive neurons. The columns and error bars represent the mean and SEM from 28 small- and 47 medium-sized DiI-positive neurons, respectively. **; *p* < 0.01 (unpaired t-test). **C**, Scatter plot of τ_fast_ against the density of TTX-R I_NaP_ (*n* = 75). The linear trend line represents the best fit using a least-squares fit (*r* = 0.69). **D**, Schematic illustration of the two-pulse protocol. TTX-R I_Na_ were induced by the first conditioning pulses (P_1_: -10 mV; 2–8,000 ms duration), which were followed by the second test pulses (P_2_: -10 mV; 50 ms duration). The second TTX-R I_Na_ was recovered with interpulse intervals of 5, 10, 20, 50, and 100 ms at a potential of -80 mV. **E.** Kinetics for the development of inactivation of TTX-R Na^+^ channels with recovery durations of 5 ms (open circles), 20 ms (closed circles), and 100 ms (open diamonds) in small- (**a**) and medium-sized (**b**) DiI-positive neurons. The P_2_/P_1_ ratio of TTX-R I_Na_ was plotted against the duration of the conditioning prepulse. The points and error bars represent the mean and SEM from eight small- and eight medium-sized DiI-positive neurons. The continuous lines represent the best fits using a double exponential function. **F.** The mean values of τ_fast_ (**a**) and A_fast_ (**b**) at different recovery durations of a two-pulse protocol in small- (open circles) and medium-sized (closed circles) DiI-positive neurons. The points and error bars represent the mean and SEM from eight small- and eight medium-sized DiI-positive neurons. Notably, τ_fast_ values were not related to the recovery duration in both types of DiI-positive neurons. **; *p* < 0.01, n.s; not significant (unpaired t-test). **G.** Schematic illustration of the two-pulse protocol. TTX-R I_Na_ were induced by the first conditioning pulse (P_1_: 2–8,000 ms duration), which was followed by the second test pulse (P_2_: 50 ms duration) with an interpulse interval of 20 ms at holding potentials of -100, -80, and -60 mV, respectively. **H.** Kinetics for the development of inactivation of TTX-R Na.^+^ channels at V_H_s of -60 mV (open circles), -80 mV (closed circles), and -100 mV (open squares) in small- (**a**) and medium-sized (**b**) DiI-positive neurons. The P_2_/P_1_ ratio of TTX-R I_Na_ was plotted against the duration of the conditioning prepulse. The points and error bars represent the mean and SEM from seven experiments for small- and medium-sized DiI-positive neurons, respectively. The continuous lines represent the best fits using a double exponential function. **I.** The mean values of τ_fast_ (**a**) and A_fast_ (**b**) at different V_H_s of a two-pulse protocol in small- (open circles) and medium-sized (closed circles) DiI-positive neurons. The points and error bars represent the mean and SEM from seven small- and seven medium-sized DiI-positive neurons. Notably, τ_fast_ values were not related to V_H_ in both types of DiI-positive neurons. *; *p* < 0.05, **; *p* < 0.01, n.s; not significant (unpaired t-test)

Both the inactivation and recovery kinetics of voltage-gated Na^+^ channels directly influence the availability of Na^+^ channels during repetitive action potentials. Therefore, the slow inactivation kinetics of TTX-R Na^+^ channels were examined using a two-pulse protocol in small- and medium-sized DiI-positive neurons (Supplementary Fig. S3A; Fig. 4Aa). The amplitude ratios of two TTX-R I_Na_ (P_2_/P_1_) were calculated and plotted against the prepulse duration for inactivation (Fig. 4Ab). The P_2_/P_1_ ratios were well-fitted to the double exponential function, which resulted in fast and slow time constants (τ_fast_ and τ_slow_) (Fig. 4Ab), suggesting that TTX-R Na^+^ channels fall into the slow inactivation category with two distinct forms of kinetics, i.e., fast and slow kinetics. There were large differences between the inactivation kinetics of small- and medium-sized DiI-positive neurons (Fig. 4Ab). τ_fast_ and τ_slow_ values were correlated with neuronal size (τ_fast_: *r* = 0.57; τ_slow_: *r* = 0.41; *n* = 41; *p* < 0.01; Fig. 4Ba and Supplementary Fig. S3C). The mean τ_fast_ values were 352.1 ± 55.3 ms (*n* = 28) and 887.8 ± 52.9 ms (*n* = 47) for small- and medium-sized DiI-positive neurons, respectively (*p* < 0.01; Fig. 4Bb). The mean τ_slow_ values were also significantly smaller in small-sized DiI-positive neurons than those in medium-sized neurons [5.6 ± 0.7 s (*n* = 28) vs. 9.9 ± 0.7 s (*n* = 47), respectively; *p* < 0.01; Supplementary Fig. S3B]. However, the amplitude fractions of τ_fast_ and τ_slow_ (A_fast_ and A_slow_, respectively) did not differ between the two DiI-positive neuron types (Supplementary Fig. S3B). Since the amplitude fraction of τ_fast_ was much larger than that of τ_slow_ (Supplementary Fig. S3B), the overall inactivation kinetics of TTX-R Na^+^ channels might be determined by τ_fast_ in both DiI-positive neuron types. τ_fast_ was further correlated with the density of TTX-R I_NaP_ in DiI-positive neurons (*r* = 0.69, *n* = 75, *p* < 0.01; Fig. 4C). However, A_fast_ and A_slow_ were not correlated with neuronal size or the density of TTX-R I_NaP_ (Supplementary Fig. S3C, D).

The extent of slow inactivation when using the two-pulse protocol depends on the duration of recovery time after the inactivation prepulse. Therefore, we examined whether inactivation kinetics were affected by changing the recovery time duration (Fig. 4D). Although the inactivation kinetics differed considerably between small- and medium-sized DiI-positive neurons (Fig. 4E), τ_fast_ values were not related to the recovery time duration in the two neuron types (Fig. 4E, F). However, A_fast_ values decreased as the recovery time duration increased (Fig. 4E, F). These results suggest that the recovery time duration after the inactivation prepulse might determine the extent rather than the kinetics of the fast component of slow inactivation. We also examined the effects of holding potentials on the inactivation kinetics of TTX-R Na^+^ channels. The two-pulse protocol was again applied to small- and medium-sized DiI-positive neurons at three different holding potentials (-60, -80, and -100 mV) (Fig. 4G). τ_fast_ was not dependent on the holding potentials in small- and medium-sized DiI-positive neurons. However, the A_fast_ values increased as the holding potentials increased (Fig. 4H, I). Thus, neither the recovery time duration nor holding potentials are responsible for the marked difference in the fast component of slow inactivation between small- and medium-sized DiI-positive neurons.

### Recovery kinetics of TTX-R Na^+^ channels in dural afferent neurons

**Fig. 5:**
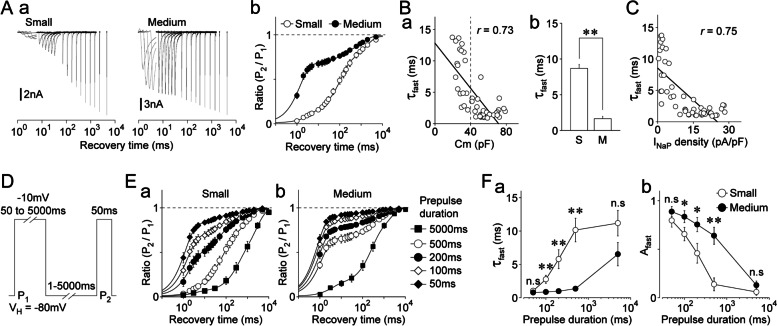
Recovery kinetics of TTX-R Na^+^ channels in dural afferent neurons. **A. a**, Typical second (P_2_) traces of TTX-R I_Na_ elicited by a two-pulse protocol in small- (left) and medium-sized (right) DiI-positive neurons. TTX-R I_Na_ were induced by the conditioning pulse (P_1_: -10 mV; 500 ms duration), which was followed by the second test pulse (P_2_: -10 mV; 50 ms duration). The second TTX-R I_Na_ was recovered with various interpulse intervals of 1–5,000 ms at a potential of -80 mV (see Supplementary Fig. S3A). **b**, Kinetics of the recovery from inactivation of TTX-R Na^+^ channels in small- (open circles) and medium-sized (closed circles) DiI-positive neurons. The P_2_/P_1_ ratio of TTX-R I_Na_ was plotted against the recovery duration. The points and error bars represent the mean and SEM from 18 small- and 33 medium-sized DiI-positive neurons. The continuous lines represent the best fits using a triple exponential function. **B. a**, Scatter plot of the fast time constant (τ_fast_) against membrane capacitance (Cm) (*n* = 51). The linear trend line represents the best fit using a least-squares fit (*r* = 0.73). **b**, The mean τ_fast_ values in small- (S) and medium-sized (M) DiI-positive neurons. The columns and error bars represent the mean and SEM from 18 small- and 33 medium-sized DiI-positive neurons. **; *p* < 0.01 (unpaired t-test). **C.** Scatter plot of the fast time constant (τ_fast_) against the density of TTX-R I_NaP_ (*n* = 51). The linear trend line represents the best fit using a least-squares fit (*r* = 0.75). **D.** Schematic illustration of the two-pulse protocol. TTX-R I_Na_ were induced by the conditioning prepulse (P_1_: -10 mV; 50 ms, 100 ms, 200 ms, and 500 ms durations), which was followed by the second test pulse (P_2_: -10 mV; 50 ms duration). The second TTX-R I_Na_ was recovered with various interpulse intervals of 1–5,000 ms at a potential of -80 mV. **E.** Kinetics of the recovery from inactivation of TTX-R Na^+^ channels with conditioning prepulse durations of 50 ms (open circles), 100 ms (closed circles), 200 ms (closed diamonds), and 500 ms (closed diamonds) in small- (**a**) and medium-sized (**b**) DiI-positive neurons. The P_2_/P_1_ ratio of TTX-R I_Na_ was plotted against the recovery duration. The points and error bars represent the mean and SEM from seven small- and eight medium-sized DiI-positive neurons. The continuous lines represent the best fits using a triple exponential function. **F.** The mean values of τ_fast_ (**a**) and A_fast_ (**b**) at different conditioning prepulse durations. The points and error bars represent the mean and SEM from seven small- and eight medium-sized DiI-positive neurons. Notably, the τ_fast_ values obtained from small-sized DiI-positive neurons were greatly reduced as the conditioning prepulse duration was shortened. *; *p* < 0.05, **; *p* < 0.01, n.s; not significant (unpaired t-test)

The extent of recovery from inactivation of TTX-R Na^+^ channels was also determined using the two-pulse protocol in small- and medium-sized DiI-positive neurons (Fig. 5A; Supplementary Fig. S4A). P_2_/P_1_ ratios were plotted against the prepulse duration for recovery times and fitted to the triple exponential function, which resulted in fast, intermediate, and slow time constants (τ_fast_, τ_intermediate_, and τ_slow_, respectively) (Fig. 5A), suggesting that TTX-R Na^+^ channels recover from inactivation with three distinct forms of kinetics, i.e., fast, intermediate, and slow recovery kinetics. The recovery kinetics of small- and medium-sized DiI-positive neurons also differed substantially (Fig. 5Ab). τ_fast_ values were correlated with the density of TTX-R I_NaP_ (*r* = 0.75, *n* = 51, *p* < 0.01; Fig. 5Ba) but τ_intermediate_ and τ_slow_ values were not (τ_intermediate_: *r* = 0.04; τ_slow_: *r* = 0.19; *n* = 51; Supplementary Fig. S3C). The mean τ_fast_ values were 8.4 ± 0.9 ms (*n* = 18) and 1.9 ± 0.2 ms (*n* = 33) for small- and medium-sized DiI-positive neurons, respectively (*p* < 0.01; Fig. 5Bb). However, the mean τ_intermediate_ and τ_slow_ values did not differ between the two neuron types (Supplementary Fig. S4B). In contrast to the inactivation kinetics, the amplitude fractions differed substantially for τ_fast_, τ_intermediate_, and τ_slow_ (A_fast_, A_intermediate_, and A_slow_, respectively) (Supplementary Fig. S4B). Taken together, the fast component of recovery from inactivation likely determines the overall recovery kinetics of TTX-R Na^+^ channels between small- and medium-sized DiI-positive neurons. τ_fast_ values were correlated with the density of TTX-R I_NaP_ (*r* = 0.75, *n* = 51, *p* < 0.01; Fig. 5C) but τ_intermediate_ and τ_slow_ values were not (Supplementary Fig. S4D). A_fast_, A_intermediate_, and A_slow_ were further correlated with neuronal size and the density of TTX-R I_NaP_ (Supplementary Fig. S4C, D).

Because recovery from inactivation should be related to the extent of inactivation, we examined whether recovery kinetics were affected by the duration of the conditioning prepulses used for channel inactivation (Fig. 5D). The extent of recovery from inactivation was greatly accelerated by shortening the inactivating prepulse duration but slowed down by lengthening this duration in small-sized, and to a lesser extent, medium-sized DiI-positive neurons (Fig. 5E). In particular, when TTX-R Na^+^ channels were inactivated with a prepulse duration of 50 or 5,000 ms, there was no difference in τ_fast_ values between the two DiI-positive neuron types [50 ms prepulse duration: 1.0 ± 0.2 ms (*n* = 9) and 0.8 ± 0.1 ms (*n* = 7) for small- and medium-sized neurons, respectively (*p* = 0.11); 5,000 ms prepulse duration: 11.2 ± 1.9 ms (*n* = 8) and 6.6 ± 1.7 ms (*n* = 8), respectively (*p* = 0.10); Fig. 5Fa]. The A_fast_ values were also increased by shortening the inactivating prepulse duration in small- and medium-sized DiI-positive neurons (Fig. 5Fb). However, when the TTX-R Na^+^ channels were inactivated with a prepulse duration of 50 or 5000 ms, there was no difference in the A_fast_ values between the two neuron types [50 ms prepulse duration: 0.80 ± 0.07 ms (*n* = 9) and 0.89 ± 0.05 (*n* = 7) for small- and medium-sized DiI-positive neurons, respectively (*p* = 0.28); 5,000 ms prepulse duration: 0.06 ± 0.03 (*n* = 8) and 0.12 ± 0.04 ms (*n* = 8), respectively (*p* = 0.09); Fig. 5Fb].

### Use-dependent inhibition of TTX-R Na^+^ channels in dural afferent neurons

The use dependence of TTX-R Na^+^ channels was also examined in small- and medium-sized DiI-positive neurons. TTX-R I_Na_ were recorded using 20 successive depolarizing test pulses (30 ms duration) from -80 mV to -10 mV every 200 ms (5 Hz) (Supplementary Fig. S5Aa). The peak amplitudes of TTX-R I_Na_ were then normalized to that elicited by the first test pulse (Supplementary Fig. S5Ab). The extent of use-dependent inactivation was larger in small-sized DiI-positive neurons than that in medium-sized neurons, where the P_20_/P_1_ ratio of TTX-R I_Na_ was 0.85 ± 0.01 (*n* = 32) and 0.94 ± 0.1 mV (*n* = 44), respectively (*p* < 0.01; Supplementary Fig. S5Bb). The P_20_/P_1_ ratio of TTX-R I_Na_ was correlated with cell capacitance (*r* = 0.62, *n* = 74, *p* < 0.01; Supplementary Fig. S5Ba) and the density of TTX-R I_NaP_ (*r* = 0.72, *n* = 74, *p* < 0.01; Supplementary Fig. S5C).

### Contribution of TTX-R I_NaP_ to the excitability of dural afferent neurons

**Fig. 6:**
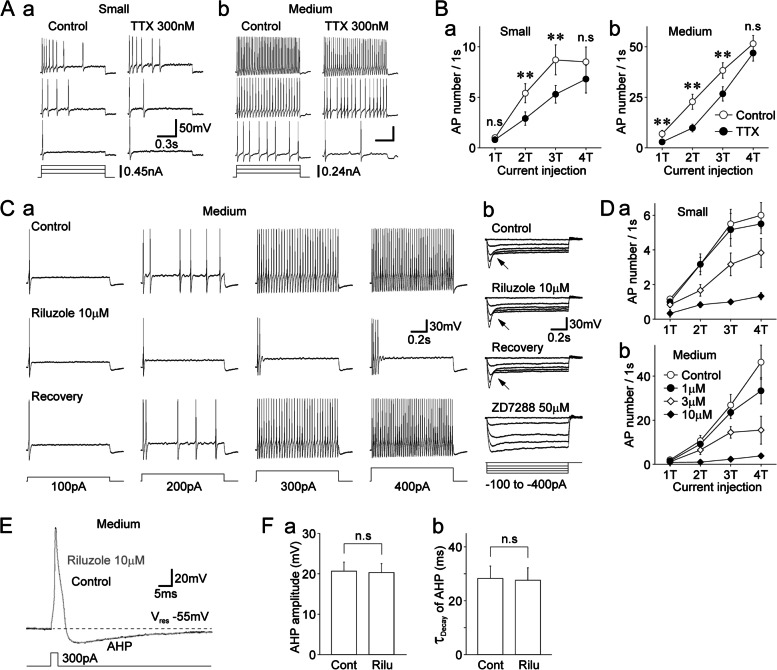
Contribution of TTX-R I_NaP_ to the excitability of dural afferent neurons.** A.** Typical voltage traces in response to depolarizing current injections in the absence (left) and presence (right) of 300 nM TTX in small- (**a**) and medium-sized (**b**) DiI-positive neurons. Three representative raw traces were elicited by one-fold threshold (1 T; 150 and 80 pA in small- and medium-sized neurons, respectively) to three-fold threshold depolarizing current injections. Phasic and tonic firing patterns are shown for small- and medium-sized DiI-positive neurons, respectively. **B.** Changes in the number of action potentials elicited by depolarizing current injections (1 T to 4 T) in the absence (open circles) and presence (closed circles) of 300-nM TTX in small- (**a**) and medium-sized (**b**) DiI-positive neurons. The points and error bars represent the mean and SEM from 25 small- and 31 medium-sized DiI-positive neurons. **; *p* < 0.01, n.s; not significant (paired t-test). **C. a**, Typical voltage traces in response to depolarizing current injections recorded before (upper), during (middle), and after (lower) the application of 10 μM riluzole. Four representative raw traces were elicited by one-fold threshold (1 T; 100 pA) to four-fold threshold (4 T; 400 pA) depolarizing current injections in medium-sized DiI-positive neurons. **b**, Typical voltage traces in response to hyperpolarizing current injections recorded before (upper), during (middle), and after (lower) the application of 10 μM riluzole in the same neuron shown in Ca. Notably, 50 μM ZD7288 but not 10 μM riluzole greatly attenuated sag potentials (arrows). **D.** Changes in the number of action potentials elicited by depolarizing current injections (1 T to 4 T) in the absence (open circles) and presence of riluzole (1, 3, and 10 μM) in small- (**a**) and medium-sized (**b**) DiI-positive neurons. The points and error bars represent the mean and SEM from six small- and six medium-sized DiI-positive neurons. **E.** Typical action potentials elicited by brief depolarizing current injections (3 ms duration, 300 pA) in the absence (black) and presence (gray) of 10 μM riluzole in medium-sized DiI-positive neurons. Note that riluzole (10 μM) did not affect the afterhyperpolarization (AHP) of action potentials. **F.** Riluzole (10 μM)-induced changes in the amplitude of AHP (**a**) and decay time constant of AHP (**b**) in medium-sized DiI-positive neurons. The columns and error bars represent the mean and SEM from six medium-sized DiI-positive neurons. n.s; not significant (paired t-test)

The above-reported results suggest that the density of TTX-R I_NaP_ might be causally related to the extent of channel inactivation during sustained membrane depolarization. Therefore, we examined whether TTX-R I_NaP_ contribute to the excitability of dural afferent neurons under current-clamp conditions. The basal membrane properties, such as resting membrane potentials and rheobase currents, which are the minimal threshold currents that trigger action potentials, are summarized in Supplementary Table S1. When the integers of threshold currents (1 T–4 T) were applied to DiI-positive neurons, phasic and tonic firing patterns were detected in most small- and medium-sized DiI-positive neurons, respectively (Fig. 6A; Supplementary Table S1), which is consistent with our previous study [24]. In the presence of TTX (300 nM), the number of action potentials triggered by depolarizing current stimuli was slightly but significantly decreased in both neuron types (Fig. 6A, B). In addition, small- and medium-sized DiI-positive neurons still exhibited phasic and tonic firing patterns, respectively, even in the presence of TTX (Fig. 6A, B). We also examined the effects of riluzole treatment on the excitability of these neurons. Riluzole treatment was found to significantly reduce the number of action potentials elicited by depolarizing current stimuli in a concentration-dependent manner. Specifically, 10 μM riluzole significantly reduced the number of action potentials elicited by 4 T stimulation in small-sized [6.0 ± 0.7 and 1.3 ± 0.2 under control and riluzole conditions, respectively (*n* = 6); *p* < 0.01] and medium-sized (46.3 ± 7.7 and 3.8 ± 0.8 under control and riluzole conditions, respectively (*n* = 6); *p* < 0.01] DiI-positive neurons (Fig. 6C, D). Hyperpolarization-activated and cyclic nucleotide-gated (HCN) channels might contribute to the tonic firing pattern observed in medium-sized DiI-positive neurons because these channels are known to contribute to neuronal excitability with regular firing properties in various central neurons [43]. However, riluzole had no inhibitory effect on the sag potentials, which were greatly decreased by the HCN channel blocker ZD7288 (50 μM) (Fig. 6Cb).

However, riluzole at micromolar concentrations is known to activate small-conductance Ca^2+^-activated K^+^ (SK_Ca_) channels [34]. Since SK_Ca_ channels contribute to afterhyperpolarization (AHP) of the action potential [44], riluzole might affect the activity of SK_Ca_ channels to reduce the frequency of action potentials in response to depolarizing current injection. Therefore, we examined whether riluzole affects AHP of action potential in DiI-positive neurons. Figure 6E shows the single action potentials elicited by brief depolarizing current stimuli (3 ms duration) in the absence and presence of 10 μM riluzole in medium-sized DiI-positive neurons. Riluzole (10 μM) had no effect on the amplitude of AHP (98.3 ± 0.8% of the control, *n* = 6, *p* = 0.08) and decay time constant of AHP (98.4 ± 4.3% of the control, *n* = 6, *p* = 0.61, Fig. 6E, F). These findings might be due to our experimental conditions, for example, the nominal Ca^2+^-free pipette solution, because the EC_50_ values of riluzole for SKCa channels are reported to be affected by the intracellular Ca^2+^ concentration [45].

### Changes in the properties of TTX-R Na^+^ channels and neuronal excitability by inflammatory mediators

**Fig. 7:**
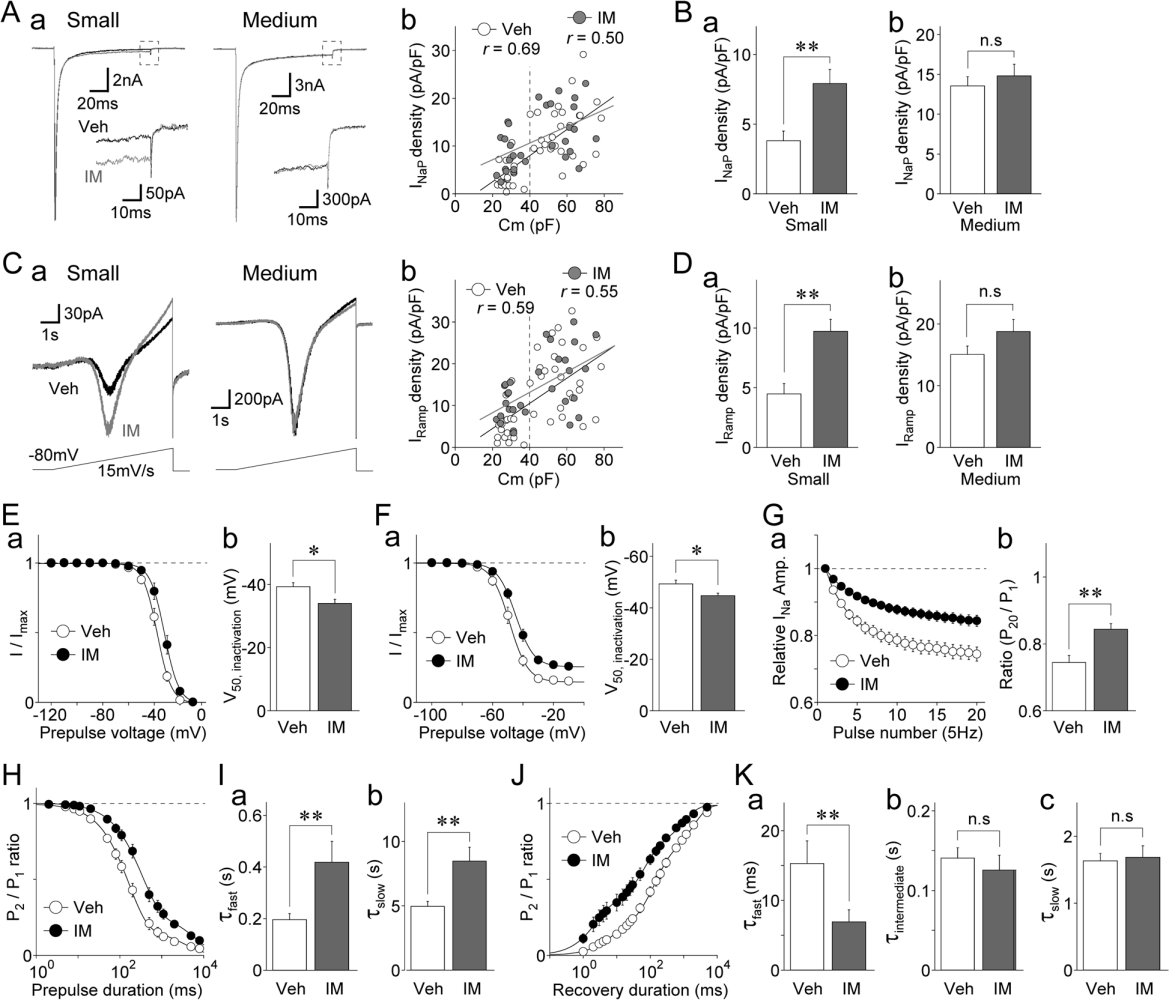
Inflammatory mediator-induced changes in the properties of TTX-R Na^+^ channels in small-sized dural afferent neurons.** A. a**, Typical traces of TTX-R I_Na_ recorded from IM-treated (gray) and vehicle-treated (black) small- (left) and medium-sized (right) DiI-positive neurons. TTX-R I_Na_ were elicited by voltage step pulses (100 ms duration; -10 mV depolarization at a V_H_ of -80 mV). Notably, TTX-R I_NaP_ were larger in the IM-treated group than those in the vehicle-treated group in small-sized DiI-positive neurons but not in medium-sized DiI-positive neurons. **b**, Scatter plot of the density of TTX-R I_NaP_ obtained from IM-treated DiI-positive neurons (gray circles; *n* = 30) and vehicle-treated DiI-positive neurons (open circles; *n* = 42) against membrane capacitance (Cm). The linear trend lines represent the best fits using a least-squares fit (IM-treated group: *r* = 0.50; vehicle-treated group: *r* = 0.69). **B.** The mean values of the current density of TTX-R I_NaP_ recorded from IM-treated and vehicle-treated small- (**a**) and medium-sized (**b**) DiI-positive neurons. The columns and error bars represent the mean and SEM from 32 (17 for vehicle-treated and 15 for IM-treated) and 40 (25 for vehicle-treated and 15 for IM-treated) neurons for small- and medium-sized DiI-positive neurons, respectively. **; *p* < 0.01, n.s; not significant (unpaired t-test). **C. a**, Typical traces of TTX-R I_Ramp_ recorded in small- (left) and medium-sized (right) DiI-positive neurons. TTX-R I_Ramp_ were elicited by voltage-ramp stimuli every 15 s (6 s duration; up to + 10 mV depolarization at a V_H_ of -80 mV; 15 mV/s). Notably, TTX-R I_Ramp_ was larger in the IM-treated group than in the vehicle-treated group in small- but not medium-sized DiI-positive neurons. **b**, Scatter plot of the density of TTX-R I_Ramp_ obtained from IM-treated DiI-positive neurons (gray circles; *n* = 30) and vehicle-treated DiI-positive neurons (open circles; *n* = 42) against membrane capacitance (Cm). The linear trend lines represent the best fits using a least-squares fit (IM-treated group: *r* = 0.55; vehicle-treated group: *r* = 0.59). **D.** The mean values of the current density of TTX-R I_Ramp_ recorded from IM- and vehicle-treated small- (**a**) and medium-sized (**b**) DiI-positive neurons. The columns and error bars represent the mean and SEM from 32 (17 for vehicle-treated and 15 for IM-treated) and 40 (25 for vehicle-treated and 15 for IM-treated) neurons for small- and medium-sized DiI-positive neurons, respectively. **; *p* < 0.01, n.s; not significant (unpaired t-test). **E. a**, Voltage-fast inactivation relationships of TTX-R Na^+^ channels in vehicle-treated (open circles) and IM-treated (closed circles) small-sized DiI-positive neurons. The points and error bars represent the mean and SEM from 16 vehicle-treated and 17 IM-treated small-sized DiI-positive neurons. The continuous lines represent the best fits using a Boltzmann function. **b**, The mean values of V_50, inactivation_ in vehicle- and IM-treated small-sized DiI-positive neurons. The columns and error bars represent the mean and SEM from 16 vehicle-treated and 17 IM-treated small-sized DiI-positive neurons. *; *p* < 0.05 (unpaired t-test). **F. a**, Voltage-slow inactivation relationships of TTX-R Na^+^ channels in vehicle-treated (open circles) and IM-treated (closed circles) small-sized DiI-positive neurons. The points and error bars represent the mean and SEM from 15 vehicle-treated and 17 IM-treated small-sized DiI-positive neurons. The continuous lines represent the best fits using a Boltzmann function. **b**, The mean values of the V_50, inactivation_ in vehicle- and IM-treated small-sized DiI-positive neurons. The columns and error bars represent the mean and SEM from 15 vehicle-treated and 17 IM-treated small-sized DiI-positive neurons. *; *p* < 0.05 (unpaired t-test). **G. a**, Time course of the amplitude of TTX-R I_Na_ during a train of 20 pulses in vehicle-treated (open circles) and IM-treated (closed circles) small-sized DiI-positive neurons. The peak amplitudes of TTX-R I_Na_ were normalized to the respective first amplitude and plotted against the pulse number. The points and error bars represent the mean and SEM from 15 vehicle-treated and 13 IM-treated small-sized DiI-positive neurons. **b**, The mean values of the P_20_/P_1_ ratio for vehicle- and IM-treated small-sized DiI-positive neurons. The columns and error bars represent the mean and SEM from 15 vehicle-treated and 13 IM-treated small-sized DiI-positive neurons. **; *p* < 0.01 (unpaired t-test). **H.** Kinetics for the development of inactivation of TTX-R Na^+^ channels in vehicle-treated (open circles) and IM-treated (closed circles) small-sized DiI-positive neurons. The P_2_/P_1_ ratio of TTX-R I_Na_ was plotted against the duration of the conditioning prepulse. The points and error bars represent the mean and SEM from 16 vehicle-treated and 19 IM-treated small-sized DiI-positive neurons. The continuous lines represent the best fits using a double exponential function. **I.** Mean values of τ_fast_ (**a**) and τ_slow_ (**b**) in vehicle- and IM-treated small-sized DiI-positive neurons. The columns and error bars represent the mean and SEM from 16 vehicle-treated and 19 IM-treated small-sized DiI-positive neurons. **; *p* < 0.01 (unpaired t-test). **J.** Kinetics of the recovery from inactivation of TTX-R Na^+^ channels in vehicle-treated (open circles) and IM-treated (closed circles) small-sized DiI-positive neurons. The P_2_/P_1_ ratio of TTX-R I_Na_ was plotted against the recovery duration. The points and error bars represent the mean and SEM from 15 vehicle-treated and 18 IM-treated small-sized DiI-positive neurons. The continuous lines represent the best fits using a triple exponential function. **K.** The mean τ_fast_ (**a**), τ_intermediate_ (**b**), and τ_slow_ (**c**) values in vehicle- and IM-treated small-sized DiI-positive neurons. The columns and error bars represent the mean and SEM from 15 vehicle-treated and 18 IM-treated small-sized DiI-positive neurons. **; *p* < 0.01, n.s; not significant (unpaired t-test)

Given that the above-reported results indicate that TTX-R I_NaP_ plays a pivotal role in the various properties of the tested channels and the excitability of DiI-positive neurons, we examined whether peripheral sensitization with IMs affected the density of TTX-R I_NaP_ and neuronal excitability. A cocktail of IMs (5-HT, PGE_2_, and bradykinin) with DiI was administered to the dura mater of living rats. After 7–10 days of this treatment, the DiI-positive neurons were used to perform an electrophysiological study (see Methods). In DiI-positive neurons isolated from Elvax-treated animals, the density of TTX-R I_NaP_ was correlated with neuronal size in both IM-treated and vehicle-treated groups, but the correlation coefficient was smaller in the IM-treated group (IM-treated group: *r* = 0.50, *n* = 30, *p* < 0.01; vehicle-treated group: *r* = 0.69, *n* = 42, *p* < 0.01; Fig. 7A). This was due to the increased density of TTX-R I_NaP_ recorded from small-sized DiI-positive neurons. Additionally, the mean density of TTX-R I_NaP_ recorded from small-sized DiI-positive neurons was significantly higher in the IM-treated group than that in the vehicle-treated group [3.8 ± 0.7 pA/pF (*n* = 17) vs. 7.9 ± 1.0 pA/pF (*n* = 15), respectively; *p* < 0.01; Fig. 7Ba]. In contrast, the density of TTX-R I_NaP_ recorded from medium-sized DiI-positive neurons did not differ significantly between the two groups [13.6 ± 1.2 pA/pF (*n* = 25) vs. 14.8 ± 1.4 pA/pF (*n* = 15), respectively; *p* = 0.50; Fig. 7Bb]. The mean density of TTX-R I_NaT_ did not differ between the IM-treated and vehicle-treated groups (data not shown). The density of TTX-R I_Ramp_ was correlated with neuronal size in both the IM-treated and vehicle-treated groups, but the correlation coefficient was smaller in the IM-treated group (IM-treated group: *r* = 0.55, *n* = 30, *p* < 0.01; vehicle-treated group: *r* = 0.59, *n* = 42, *p* < 0.01; Fig. 7C). The mean density of TTX-R I_Ramp_ recorded from small-sized DiI-positive neurons was significantly higher in the IM-treated group than that in the vehicle-treated group (4.5 ± 0.9 pA/pF (*n* = 17) vs. 9.7 ± 1.0 pA/pF (*n* = 15), respectively; *p* < 0.01; Fig. 7Da). However, the density of TTX-R I_Ramp_ recorded from medium-sized DiI-positive neurons did not differ statistically between the groups (*p* = 0.12; Fig. 7Db). Taken together, these results suggest that the noninactivating components of TTX-R I_Na_ were enhanced in small-sized DiI-positive neurons by peripheral sensitization due to IMs.

An increase in the density of TTX-R I_NaP_ is expected to alter the various properties of these channels in IM-treated small-sized DiI-positive neurons, as shown in the results reported above. Therefore, we also examined the voltage dependence, use-dependent inhibition, and inactivation and recovery kinetics of TTX-R Na^+^ channels in the IM-treated and vehicle-treated groups. We found that various properties of TTX-R Na^+^ channels were affected in small-sized but not medium-sized DiI-positive neurons (Fig. 7 and Supplementary Fig. S6). Although the V_50_ values for activation did not differ between the groups, the V_50_ values for fast inactivation were significantly shifted to the depolarizing range, where the mean V_50_ values for fast inactivation obtained from small-sized DiI-positive neurons were -34.0 ± 1.3 mV (*n* = 17) and -39.3 ± 1.2 mV (*n* = 16) in IM-treated and vehicle-treated groups, respectively (*p* < 0.01; Fig. 7E). The mean V_50_ values for slow inactivation obtained from small-sized DiI-positive neurons were also significantly higher in the IM-treated group compared to those in the vehicle-treated group [-44.7 ± 0.9 mV (*n* = 17) vs. -49.3 ± 1.5 mV (*n* = 15), respectively; *p* < 0.01; Fig. 7F]. The extent of use-dependent inhibition was reduced in the IM-treated group compared to that in the vehicle-treated group [P_20_/P_1_ ratio: 0.84 ± 0.02 for the IM-treated group (*n* = 13) vs. 0.74 ± 0.02 for the vehicle-treated group (*n* = 15); *p* < 0.01; Fig. 7G]. In addition, the inactivation observed from small-sized DiI-positive neurons developed significantly more slowly in the IM-treated group than in the vehicle-treated group. The τ_fast_ values were 418.2 ± 81.3 ms (*n* = 19) and 195.9 ± 23.5 ms (*n* = 16) in the IM-treated and vehicle-treated groups, respectively (*p* < 0.01; Fig. 7H, Ia); the τ_slow_ values were also larger in the IM-treated and vehicle-treated groups, respectively (*p* < 0.01, Fig. 7Ib). Furthermore, the extent of recovery from inactivation for small-sized DiI-positive neurons occurred significantly more quickly in the IM-treated group than in the vehicle-treated group; the τ_fast_ values were 6.9 ± 1.3 ms (*n* = 18) and 15.3 ± 3.2 ms (*n* = 15) in the IM-treated and vehicle-treated groups, respectively (*p* < 0.01; Fig. 7J, Ka). However, there was no difference in the τ_intermediate_ and τ_slow_ values between the IM-treated and vehicle-treated groups (Fig. 7Kb, Kc). In contrast, in medium-sized DiI-positive neurons, we detected no differences in voltage dependence, the onset of inactivation, and recovery from inactivation of TTX-R Na^+^ channels between the IM-treated and vehicle-treated groups (Supplementary Fig. S6).

**Fig. 8:**
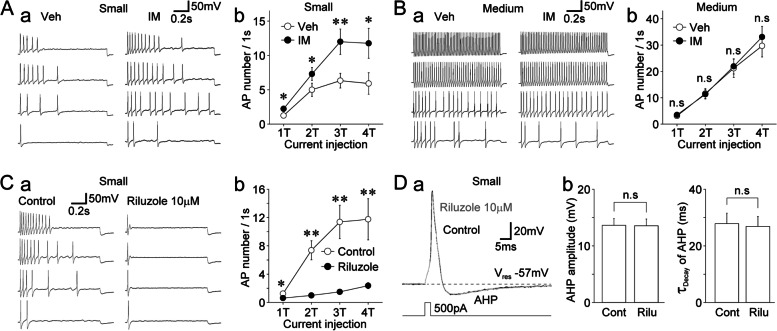
Inflammatory mediator-induced changes in the excitability of small-sized dural afferent neurons. **A. a**, Typical voltage traces in response to depolarizing current injections in vehicle-treated (left) and IM-treated (right) small-sized DiI-positive neurons. Four representative raw traces were elicited by one-fold threshold (1 T; 150 pA) to four-fold threshold depolarizing current injections. **b**, Changes in the number of action potentials elicited by depolarizing current injections (1 T–4 T) in vehicle-treated (open circles) and IM-treated (closed circles) small-sized DiI-positive neurons. The points and error bars represent the mean and SEM from 12 vehicle-treated and 13 IM-treated small-sized DiI-positive neurons. *; *p* < 0.05, **; *p* < 0.01 (unpaired t-test). **B. a**, Typical voltage traces in response to depolarizing current injections in vehicle-treated (left) and IM-treated (right) medium-sized DiI-positive neurons. Four representative raw traces were elicited by one-fold threshold (1 T; 100 pA) to four-fold threshold depolarizing current injections. **b**, Changes in the number of action potentials elicited by depolarizing current injections (1 T–4 T) in vehicle-treated (open circles) and IM-treated (closed circles) medium-sized DiI-positive neurons. The points and error bars represent the mean and SEM from 10 vehicle-treated and 12 IM-treated medium-sized DiI-positive neurons. n.s; not significant (unpaired t-test). **C. a**, Typical voltage traces in response to depolarizing current injections in IM-treated small-sized DiI-positive neurons in the absence (left) and presence (right) of 10 μM riluzole. Four representative raw traces were elicited by one-fold threshold (1 T; 120 pA) to four-fold threshold depolarizing current injections. **b**, Changes in the number of action potentials elicited by depolarizing current injections (1 T–4 T) in IM-treated small-sized DiI-positive neurons in the absence (left) and presence (right) of 10 μM riluzole. The points and error bars represent the mean and SEM from eight IM-treated small-sized DiI-positive neurons. *; *p* < 0.05, **; *p* < 0.01 (paired t-test). **D. a**, Typical action potentials elicited by brief depolarizing current injections (3 ms duration, 500 pA) in the absence (black) and presence (gray) of 10 μM riluzole in medium-sized DiI-positive neurons. Note that riluzole (10 μM) did not affect afterhyperpolarization (AHP) of action potentials. **b**, Riluzole (10 μM)-induced changes in the amplitude of AHP (left) and decay time constant of AHP (right) in IM-treated small-sized DiI-positive neurons. The columns and error bars represent the mean and SEM from six IM-treated small-sized DiI-positive neurons. n.s; not significant (paired t-test)

Finally, we examined whether the excitability was changed in IM-treated DiI-positive neurons. The basal membrane properties of DiI-positive neurons in the vehicle-treated and IM-treated groups are summarized in Supplementary Table S2. While the firing patterns of small- or medium-sized DiI-positive neurons did not differ between the vehicle-treated and IM-treated groups, the rheobase currents measured in small-sized DiI-positive neurons were significantly lower in the IM-treated group than in the vehicle-treated group (Supplementary Table S2). In the presence of TTX (300 nM), action potentials were elicited by depolarizing current stimuli (1 T–4 T) in DiI-positive neurons of the vehicle-treated and IM-treated groups. In small-sized DiI-positive neurons, the number of action potentials was higher in the IM-treated group than in the vehicle-treated group (Fig. 8A). However, in medium-sized DiI-positive neurons, there was no difference in the number of action potentials observed between the vehicle-treated and IM-treated groups (Fig. 8B). We further examined the effects of riluzole on the excitability of IM-treated small-sized DiI-positive neurons. Riluzole (10 μM) greatly reduced the number of action potentials elicited by depolarizing current stimuli, in which it reduced the number of action potentials elicited by 4 T stimulation (11.8 ± 2.9 and 2.4 ± 0.4 under control and riluzole conditions, respectively, *n* = 8, *p* < 0.01, Fig. 8C). When single action potentials were elicited by brief depolarizing current stimuli (3 ms duration) in IM-treated small-sized DiI-positive neurons, riluzole (10 μM) had no effect on the amplitude of AHP (99.4 ± 1.0% of the control, *n* = 6, *p* = 0.48) and decay time constant of AHP (96.8 ± 3.6% of the control, *n* = 6, *p* = 0.45, Fig. 8D).

## Discussion

### Contribution of TTX-R I_NaP_ to the excitability of dural afferent neurons

In the present study, the density of TTX-R I_NaP_ and TTX-R I_Ramp_ was correlated with the size of DiI-positive neurons. Both TTX-R I_NaP_ and TTX-R I_Ramp_ were sensitive to riluzole and A803467 (a specific Na_V_1.8 blocker), indicating that these currents were mediated by the same channels. Such noninactivating currents might correspond to the I_NaP_ found in DRG neurons [21] as well as the central neurons in various brain regions [11, 46–49]. The contamination of other TTX-R Na^+^ channel subtypes, such as Na_V_1.9 or Na_V_1.5, might be negligible because the V_50,activation_ and V_50,inactivation_ values for such subtypes have ranges that include relatively hyperpolarized potentials [18, 50]. In addition, since the voltage-fast inactivation relationships of TTX-R I_NaT_ and TTX-R I_NaP_ were largely the same, TTX-R I_NaP_ is mediated by Na_V_1.8 rather than other subtypes. Consistent with these results, we also found that the voltage dependences of TTX-R Na^+^ channels, such as the activation and instantaneous steady-state fast inactivation relationships, did not differ among DiI-positive neurons and that relationships did not exist between the voltage dependences and neuronal size or the density of TTX-R I_NaP_. However, there were marked differences in the fast component (i.e., τ_fast_) of slow inactivation and in recovery kinetics of small- and medium-sized DiI-positive neurons. The τ_fast_ values for the onset of slow inactivation were significantly lower in small-sized DiI-positive neurons than in medium-sized neurons, and, importantly, these values were correlated with the density of TTX-R I_NaP_ and, to a lesser extent, neuronal size. Similarly, the τ_fast_ values for recovery from inactivation were significantly higher in small-sized DiI-positive neurons than those in medium-sized neurons, and these values were also correlated with the density of TTX-R I_NaP_. Thus, TTX-R I_NaP_ are apparently involved in channel properties such as slow inactivation and recovery kinetics.

In the current study, changes to the holding potential and recovery duration did not affect the τ_fast_ values in both small- and medium-sized DiI-positive neurons. Because large differences in τ_fast_ values existed between small- and medium-sized DiI-positive neurons, the holding potential and/or recovery duration might not be responsible for the fundamental difference in the fast component of inactivation kinetics. Observed changes in A_fast_ values suggest that the holding potential and recovery duration may affect the extent rather than the rate of slow inactivation. We also found that the τ_fast_ values for recovery kinetics were greatly decreased or increased by shortening or lengthening the duration of the inactivating prepulse, respectively, in small- and medium-sized DiI-positive neurons, suggesting that the extent of slow inactivation during depolarizing prepulses might determine the recovery kinetics of TTX-R Na^+^ channels in these neurons. Therefore, differences in the slow inactivation of TTX-R Na^+^ channels are likely to result in marked differences in recovery kinetics and use-dependent inhibition, as shown by other researchers [9, 10]. Notably, the extent of use-dependent inhibition and kinetics for slow inactivation and recovery from inactivation of TTX-R Na^+^ channels were more strongly correlated with the density of TTX-R I_NaP_ than with cell capacitance, suggesting that a relationship potentially exists between TTX-R I_NaP_ and channel functions.

Growing evidence supports the important role played by I_NaP_ in the repetitive firing of action potentials in response to sustained membrane depolarization in central neurons [11, 51–56]. In addition, the amplitude of I_NaP_ is known to be related to the firing patterns of spinal neurons [57]. Human Na_V_1.8 produces larger persistent currents and ramp currents than those produced by rat Na_V_1.8, and these properties of Na_V_1.8 currents are related to the increased firing frequency in neurons [21]. However, the functional role of TTX-R I_NaP_ in nociceptive neurons remains largely unknown. In our study, the firing patterns and number of action potentials in response to depolarizing current stimuli differed considerably between the two tested types of DiI-positive neurons. Given that firing patterns were not changed even in the presence of TTX and that riluzole greatly reduced the extent of the repetitive generation of action potentials in small- and medium-sized DiI-positive neurons, riluzole-sensitive TTX-R I_NaP_ might play a major role in the repetitive generation of action potentials in C-type dural afferent neurons.

### Possible factors involved in the differential density of TTX-R I_NaP_

Several factors may be responsible for the differential density of TTX-R I_NaP_ and inactivation kinetics in C-type dural afferent neurons. For example, the incorporation of accessory β subunits into various subtypes of Na^+^ channels is known to affect I_NaP_ and inactivation kinetics [58]. In a previous study using *Scn1b*^−/−^ mice, β1 subunits contributed to larger TTX-R I_NaP_ and faster recovery from inactivation in small DRG neurons [59]. However, since a marked difference in the inactivation kinetics between *Scn1b*^−/−^ and *Scn1b*^+/+^ mice was not detected [59], β1 subunits alone are unlikely to be responsible for the different TTX-R Na^+^ channel properties among dural afferent neurons. Alternatively, splice variants for Na_V_1.8 [60, 61] might explain the marked differences in the TTX-R Na^+^ channel properties of small- and medium-sized DiI-positive neurons. However, the obvious impact of splicing events on the function of Na_V_1.8 has yet to be found [61]. Thus, further research is required to reveal which factors are involved in the differential properties of TTX-R Na^+^ channels in C-type dural afferent neurons. Nevertheless, previous studies have demonstrated the differential slow inactivation and use-dependent inhibition of Na_V_1.8 in two subpopulations of small-sized DRG neurons, i.e., isolectin B_4_-positive nonpeptidergic and IB_4_-negative peptidergic neurons [10]. Furthermore, the protein calmodulin, which is reportedly involved in the TTX-R Na^+^ channel differs between these two subpopulations [62]. However, in the present study, most small- and medium-sized DiI-positive neurons were likely to be peptidergic neurons because ~ 80% of these neurons express CGRP [23], which is regarded as a peptidergic neuron marker [63]. It would be of interest to examine whether calmodulin is also involved in the differential properties of TTX-R Na^+^ channels in C-type dural afferent neurons.

### TTX-R I_NaP_ as pharmacological targets for migraine medication

The increased neuronal excitability of nociceptive neurons is closely related to inflammatory hyperalgesia, i.e., IM treatment increases the number of action potentials elicited by depolarizing current stimuli in nociceptive neurons [23, 25]. In this regard, Na_V_1.8 expressed in nociceptive neurons are reportedly subject to inflammatory sensitization because these channels are positively modulated by IMs [64, 65]. Furthermore, CGRP-induced neurogenic inflammation and the subsequent sensitization of dural afferent neurons play a pivotal role in migraine pathology [66, 67]. In the present study, we found that the density of TTX-R I_NaP_ was significantly larger in small-sized DiI-positive neurons treated with IM than those treated with a vehicle. This increase in TTX-R I_NaP_ density could lead to changes in channel properties, including the inactivation and recovery kinetics, which might eventually affect the firing properties and/or frequency of action potentials in relation to depolarizing stimuli. Interestingly, mutations in Na_V_1.1, which are associated with familial hemiplegic migraine type 3, cause an increase in the density of I_NaP_, a defect in the inactivation process, and repetitive generation of action potentials [68]. Although the mechanisms that underlie the IM-induced increase in TTX-R I_NaP_ remain to be elucidated, our study suggests that TTX-R I_NaP_ are involved in peripheral sensitization mediated by neurogenic inflammation around the dura mater. However, in medium-sized DiI-positive neurons, the density of TTX-R I_NaP_ and the properties of TTX-R Na^+^ channels did not differ between the IM-treated and control groups. Therefore, further studies are required to determine the role of medium-sized DiI-positive neurons in the inflammatory sensitization of dural afferents.

Voltage-gated Na^+^ channels may be related to migraine pathology, given that most of the effective drugs used for migraine prophylaxis, such as β-blockers (e.g., propranolol), tricyclic antidepressants (e.g., amitriptyline), Ca^2+^ channel blockers (e.g., flunarizine), and Na^+^ channel blockers (e.g., valproic acid) [69, 70], are known to inhibit voltage-gated Na^+^ channels [71–74]. Although there is no direct evidence that TTX-R Na^+^ channels and TTX-R I_NaP_ are involved in migraine pathology, our previous studies have also shown that either amitriptyline or propranolol preferentially inhibit TTX-R I_NaP_ and decrease the excitability of dural afferent neurons [75, 76]. It would be interesting to investigate whether CGRP-mediated neurogenic inflammation affects the properties of TTX-R Na^+^ channels and TTX-R I_NaP_ and whether drugs used to treat and prevent migraine attacks can inhibit TTX-R I_NaP_ in dural afferent neurons.

In the present study, we shed light on the role of Na_V_1.8-mediated I_NaP_ in the excitability of dural afferent neurons. However, nociceptive neurons also express another TTX-R Na^+^ channel subtype, Na_V_1.9 [18.19]. Na_V_1.9 has been implicated in inflammatory hyperalgesia induced by IMs [77–79], and Na_V_1.9-mediated currents are potentiated by IMs [20]. Interestingly, a recent study reported that with the abnormal activation of Na_V_1.9, the TTX-R Na^+^ channels subtypes expressed in nociceptive neurons by nitric oxide are responsible for medication-overuse headaches induced by triptan migraine medicine [80]. Since Na_V_1.9-mediated currents, including slow voltage ramp-induced currents, were recorded from most small- and medium-sized DiI-positive neurons, it would be of great interest to examine whether Na_V_1.9-mediated I_Ramp_ is also involved in the IM-mediated increase in the excitability of small-sized DiI-positive neurons.

## Conclusion

In conclusion, we found that the density of TTX-R I_NaP_ differed considerably among C-type dural afferent neurons and that this difference was correlated with the various properties of TTX-R Na^+^ channels, including inactivation and recovery kinetics, which might contribute to the differential excitability of these neurons. We also found that chronic IM treatment applied to the dura mater caused an increase in the density of TTX-R I_NaP_ in small-sized DiI-positive neurons, which in turn affected the various properties of TTX-R Na^+^ channels, suggesting that TTX-R I_NaP_ are involved in the peripheral sensitization of a subset of dural afferent neurons by IMs.

## Supplementary Information

**Additional file 1:** **Supplementary Fig. S1. **Basal properties of TTX-RI_NaP_ and I_Ramp_ in adult male and female rats. **A.** Typical traces of TTX-R I_NaP_(**a**) and I_Ramp_ (**b**) in the absence and presence of 0.1% DMSO (v/v). Similar results were obtained from five independent experiments. **B.** Scatter plots of the density of TTX-R I_NaP_ against membranecapacitance (Cm) obtained from DiI-positive neurons derived from young male (**a**, *n* = 71 neurons, same to Fig. 1D), adult male (**b**, *n* = 62 neurons), and adult female rats (**c**, *n* = 60neurons). The linear trend lines represent the best fit using a least-squaresfit (**a**; *r* = 0.65, **b**; *r* = 0.73, **c**; *r* = 0.76). **C.** The mean values of the density of TTX-R I_NaP_ in small-sized (**a**; *n* = 31 neurons for young male, *n* = 28 neurons for adult male, and n = 31 neurons for adult female rats) and medium-sized (**b**; *n* = 40 neurons for young male, n = 34 neurons for adult male, and n = 29 neurons for adult female rats) DiI-positive neurons. The columns and error bars represent the mean and SEM. n.s; not significant (unpaired t-test). **D.** Scatter plots of the density of TTX-R I_Ramp_ against membrane capacitance (Cm) obtained from DiI-positive neurons derived from young adult male (**a**, *n* = 152 neurons, same to Fig. 2Da), adult male (**b**, *n* = 62 neurons), and adult female rats (**c**,*n* = 60 neurons). The linear trend lines represent the best fit using aleast-squares fit (**a**; *r* = 0.47, **b**; *r* = 0.69, **c**; *r*= 0.76). **E.** The mean values of the density of TTX-R I_Ramp_ in small-sized (**a**; *n* = 60 neurons for young male, *n* = 28 neurons for adult male, and *n* = 31 neurons for adult female rats) and medium-sized (**b**; *n* = 92 neurons for young male, *n* = 34 neurons for adult male, and *n* = 29 neurons for adult female rats) DiI-positive neurons. The columns and errorbars represent the mean and SEM. n.s; not significant (unpaired t-test). **Supplementary Fig. S2. V**oltage dependence of TTX-R Na^+^ channels in dural afferent neurons. **A.** **a**, Typical traces of TTX-R I_Na_ elicited by step pulses (100 msdepolarization pulses from -80 to +30 mV in 10 mV increments at a V_H _of -80 mV) in small- (left) and medium-sized (right) DiI-positive neurons. **b**, Conductance-voltage relationships of TTX-R Na^+^channels in small- (open circles) and medium-sized (closed circles) DiI-positive neurons. The points and error bars represent the mean and SEM from 32 small-sized and 44 medium-sized DiI-positive neurons. The continuous lines represent the best fits using a Boltzmann function. **B. **Scatter plot of the half-maximal voltage for activation (V_50, activation_) against membrane capacitance (Cm) (*n* = 76). The linear trend line represents the best fit using a least-squares fit (*r *= 0.01). **C. **Scatter plot of the half-maximal voltage for activation (V_50, activation_) against the density of TTX-R I_NaP_ (*n* = 76). The linear trend line represents the best fit using a least-squares fit (*r* = 0.02). **D.** Typical traces of TTX-R I_Na_ elicited by step pulses (100 ms duration; up to -50 mV in 10 mV increments) at V_H_s of -80 mV (**a**) or -120 mV (**b**) in the same small- (left) and medium-sized (right) DiI-positive neurons. Notably, these slowly desensitizing I_Na_, which were mediated by Na_V_1.9, were elicited when neurons were held at a V_H_ of -120 mV but not of -80 mV. **E.** Current-voltage relationships of TTX-R Na^+ ^channels in small- (left) and medium-sized (right) DiI-positive neurons. The points and error bars represent the mean and SEM from eight small-sized and seven medium-sized DiI-positive neurons. **F.** **a**, Schematic illustration of the voltage step stimulation for steady-state fast inactivation of TTX-R Na^+^ channels. **b**, Typical traces of TTX-R I_Na_ elicited by step pulses in medium-sized DiI-positive neurons. The inset represents the I_NaP_ region (dotted box) with an expanded time scale. **c**, Current-voltage relationships of TTX-R I_NaT _(open circles) and I_NaP_ (closed circles) in medium-sized DiI-positive neurons. Each point represents the mean and SEM from 10experiments. The continuous lines represent the best fits using a Boltzmann function. **G. **Midpoint voltage for inactivation (V_50, inactivation_, **a**) and slope factor (**b**) of TTX-R I_NaT_ and I_NaP_. The columns and error bars represent the mean and SEM from 10 medium-sized DiI-positive neurons. n.s; not significant (paired t-test). **Supplementary Fig. S3. **Kinetic parameters for the development of inactivation of TTX-R Na^+^ channels in dural afferent neurons. **A.** Schematic illustration of the two-pulse protocols used for the development of inactivation of TTX-R Na^+ ^channels. TTX-R I_Na_ were induced by the conditioning prepulse (P_1_: -10 mV; 2–8,000 ms duration), which was followed by the test pulse (P_2_: -10 mV; 50 ms duration). The second TTX-R I_Na_ was recovered with an interpulse interval of 20 ms at a V_H_ of -80 mV. **B.** The mean values of τ_slow_ (**a**), A_fast_ (**b**), and A_slow_ (**c**) in small- (S) and medium-sized (M) DiI-positive neurons. The columns and error bars represent the mean and SEM from 28 small- and 47 medium-sized DiI-positive neurons. **; *p* < 0.01, n.s; notsignificant (unpaired t-test). **C. **Scatter plots of τ_slow_ (**a**), A_fast_ (**b**), and A_slow_(**c**) against membrane capacitance (Cm) (*n* = 75). The linear lines represent the best fits using a least-squaresfit. **D. **Scatter plots of τ_slow_ (**a**), A_fast_ (**b**), and A_slow _(**c**) against the density of TTX-R I_NaP _(*n* = 75). The linear lines represent the best fits using a least-squares fit. **Supplementary Fig. S4. **Kinetic parameters for the recovery from inactivation of TTX-R Na^+^ channels in dural afferent neurons. **A.** Schematic illustration of the two-pulse protocol used for the recovery from inactivation of TTX-R Na^+^ channels. TTX-R I_Na_ were induced by the conditioning prepulse (P_1_: -10 mV; 500 ms duration), which was followed by the test pulse (P_2_: -10 mV; 50msduration). The second TTX-R I_Na_ was recovered with various interpulse intervals of 1–5,000 ms at a V_H_ of -80 mV. **B.** The mean values of τ_intermediate_ (**a**), τ_slow_ (**b**), A_fast_ (**c**), A_intermediate_(**d**), and A_slow_ (**e**) in small- (S) and medium-sized (M) DiI-positive neurons. The columns and error bars represent the mean and SEM from 18 small-sized and 33 medium-sized DiI-positive neurons. **; *p* < 0.01, n.s; notsignificant (unpaired t-test). **C. **Scatter plots of τ_intermediate_ (**a**), τ_slow_ (**b**), A_fast _(**c**), A_intermediate_ (**d**), and A_slow_ (**e**) against membrane capacitance (Cm) (*n*= 51). The linear trend lines represent the best fits using a least-squaresfit. **D. **Scatter plots of τ_intermediate_ (**a**), τ_slow_ (**b**), A_fast _(**c**), A_intermediate_ (**d**), and A_slow_ against the density of TTX-R I_NaP_ (*n* = 51). The linear trend lines represent the best fits using a least-squares fit. **Supplementary Fig. S5. **Use-dependency of TTX-R Na^+^ channels in dural afferent neurons. **A.** **a**, Typical traces of TTX-R I_Na_ elicited by 20 successive voltage step pulses (5 Hz; -10mV; 30 ms duration) in small- (left) and medium-sized (right) DiI-positive neurons. **b**, Time course of the amplitude of TTX-R I_Na_ during a train of 20 pulses in small- (open circles) and medium-sized (closed circles) DiI-positive neurons. The peak amplitudes of TTX-R I_Na_ were normalized to the respective first amplitude and plotted against the pulse number. The points and error bars represent the mean and SEM from 32 small- and 44 medium-sized DiI-positive neurons. **B.** **a**, Scatter plot of the P_20_/P_1_ ratio against membrane capacitance (Cm) (*n* = 76). The linear trend line represents the best fit using a least-squares fit (*r* = 0.62). **b**, The mean values of the P_20_/P_1 _ratio in small- (S) and medium-sized (M) DiI-positive neurons. The columns and error bars represent the mean and SEM from 32 small- and 44 medium-sized DiI-positive neurons. **; *p* < 0.01 (unpaired t-test). **C. **Scatter plot of the P_20_/P_1_ ratio against the density of TTX-R I_NaP_ (*n* = 76). The linear trend line represents the best fitusing a least-squares fit (*r* = 0.72). **Supplementary Fig. S6. **Inflammatory mediator-induced changes in the properties of TTX-R Na^+ ^channels in medium-sized dural afferent neurons. **A**. **a**, Voltage-fast inactivation relationships of TTX-R Na^+^channels in vehicle-treated (open circles) and IM-treated (closed circles) medium-sized DiI-positive neurons. Each point represents the mean and SEM from 25 vehicle-treated and 18 IM-treated medium-sized DiI-positive neurons. The continuous lines represent the best fits using a Boltzmann function. **b**, The mean values of the V_50,inactivation_ in vehicle- and IM-treated medium-sized DiI-positive neurons. The columns and error bars represent the mean and SEM from 25 vehicle-treated and 18 IM-treated medium-sized DiI-positive neurons. n.s; not significant (unpaired t-test). **B.** **a**, Voltage-slow inactivation relationships of TTX-R Na^+ ^channels in vehicle-treated (open circles) and IM-treated (closed circles) medium-sized DiI-positive neurons. The points and error bars represent the mean and SEM from 21 vehicle-treated and 18 IM-treated medium-sized DiI-positive neurons. The continuous lines represent the best fits using a Boltzmann function. **b**, The mean values of V_50, inactivation_ in vehicle- and IM-treated medium-sized DiI-positive neurons. The bars and errors represent the mean and SEM from 21 vehicle-treated and 18 IM-treated medium-sized DiI-positive neurons. n.s; not significant (unpaired t-test). **C.** **a**, Time course of the amplitude of TTX-R I_Na_ during a train of 20 pulses in vehicle-treated (open circles) and IM-treated (closed circles) medium-sized DiI-positive neurons. The peak amplitudes of TTX-R I_Na_ were normalized to the respective first amplitude and plotted against the pulse number. The points and error bars represent the mean and SEM from 23 vehicle-treated and 14 IM-treated medium-sized DiI-positive neurons. **b**, The mean values of the P_20_/P_1_ ratio for vehicle- and IM-treated medium-sized DiI-positive neurons. The columns and error bars represent the mean and SEM from 23 vehicle-treated and 14 IM-treated medium-sized DiI-positive neurons. *; *p* < 0.05 (unpairedt-test). **D.** Kinetics for the development of inactivation of TTX-R Na^+^ channels in vehicle-treated (opencircles) and IM-treated (closed circles) medium-sized DiI-positive neurons. The P_2_/P_1_ ratio of TTX-R I_Na_ was plotted against the duration of the conditioning prepulse. The points and error bars represent the mean and SEM from 25 vehicle-treated and 18 IM-treated medium-sized DiI-positive neurons. The continuous lines represent the best fits using a double exponential function. **E.** The mean values of τ_fast _(**a**) and τ_slow_ (**b**) in vehicle- and IM-treated medium-sized DiI-positive neurons. The columns and error bars represent the mean and SEM from 25 vehicle-treated and 18 IM-treated medium-sized DiI-positive neurons. n.s; not significant (unpairedt-test). **F.** Kinetics of the recovery from inactivation of TTX-R Na^+^ channels in vehicle-treated (open circles) and IM-treated (closed circles) medium-sized DiI-positive neurons. The P_2_/P_1_ ratio of TTX-R I_Na_ was plotted against the recovery duration. The points and error bars represent the mean and SEM from 25 vehicle-treated and 18 IM-treated medium-sized DiI-positive neurons.The continuous lines represent the best fits using a triple exponential function. **G.** The mean τ_fast_ (**a**), τ_intermediate_ (**b**), and τ_slow_ (**c**) values in vehicle- and IM-treated medium-sized DiI-positive neurons. The columns and error bars represent the mean and SEM from 25 vehicle-treated and 18 IM-treated medium-sized DiI-positive neurons. n.s; not significant (unpairedt-test). **Supplementary Table S1. **Basal membrane properties of C-type dural afferent neurons.** Supplementary Table S2. **Basal membrane properties of vehicle-treated and IM-treated C-type dural afferent neurons. 

## Data Availability

The datasets used and/or analysed during the current study are available from the corresponding author on reasonable request.

## Abbreviations

DRG: Dorsal root ganglia
IM: Inflammatory mediator
I_NaP_: Persistent Na^+^ currents
I_NaT_: Transient Na^+^ currents
I_Ramp_: Voltage ramp-induced Na^+^ currents
PGE_2_: Prostaglandin E_2_
SEM: Standard error of the mean
SK_Ca_ channels: Small-conductance Ca^2+^-activated K^+^ channels
TG: Trigeminal ganglia
TTX-R: Tetrodotoxin-resistant
TTX-S: Tetrodotoxin-sensitive
