# Supplementary material for: Contribution of tetrodotoxin-resistant persistent Na+ currents to the excitability of C-type dural afferent neurons in rats
Source: J Headache Pain. 2022 Jun 28;23(1):73. doi: 10.1186/s10194-022-01443-7 (PMC9238149; doi:10.1186/s10194-022-01443-7)
Supplement: Supplementary file 1 — Additional file 1: Supplementary Fig. S1. Basal properties of TTX-RINaP and IRamp in adult male and female rats. A. Typical traces of TTX-R INaP(a) and IRamp (b) in the absence and presence of 0.1% DMSO (v/v). Similar results were obtained from five independent experiments. B. Scatter plots of the density of TTX-R INaP against membranecapacitance (Cm) obtained from DiI-positive neurons derived from young male (a, n = 71 neurons, same to Fig. 1D), adult male (b, n = 62 neurons), and adult female rats (c, n = 60neurons). The linear trend lines represent the best fit using a least-squaresfit (a; r = 0.65, b; r = 0.73, c; r = 0.76). C. The mean values of the density of TTX-R INaP in small-sized (a; n = 31 neurons for young male, n = 28 neurons for adult male, and n = 31 neurons for adult female rats) and medium-sized (b; n = 40 neurons for young male, n = 34 neurons for adult male, and n = 29 neurons for adult female rats) DiI-positive neurons. The columns and error bars represent the mean and SEM. n.s; not significant (unpaired t-test). D. Scatter plots of the density of TTX-R IRamp against membrane capacitance (Cm) obtained from DiI-positive neurons derived from young adult male (a, n = 152 neurons, same to Fig. 2Da), adult male (b, n = 62 neurons), and adult female rats (c,n = 60 neurons). The linear trend lines represent the best fit using aleast-squares fit (a; r = 0.47, b; r = 0.69, c; r= 0.76). E. The mean values of the density of TTX-R IRamp in small-sized (a; n = 60 neurons for young male, n = 28 neurons for adult male, and n = 31 neurons for adult female rats) and medium-sized (b; n = 92 neurons for young male, n = 34 neurons for adult male, and n = 29 neurons for adult female rats) DiI-positive neurons. The columns and errorbars represent the mean and SEM. n.s; not significant (unpaired t-test). Supplementary Fig. S2. Voltage dependence of TTX-R Na+ channels in dural afferent neurons. A. a, Typical traces of TTX-R INa elicited by step pulses (100 msdepolariza [file 10194_2022_1443_MOESM1_ESM.docx]

**Supplementary** **materials**

**Figure S1**


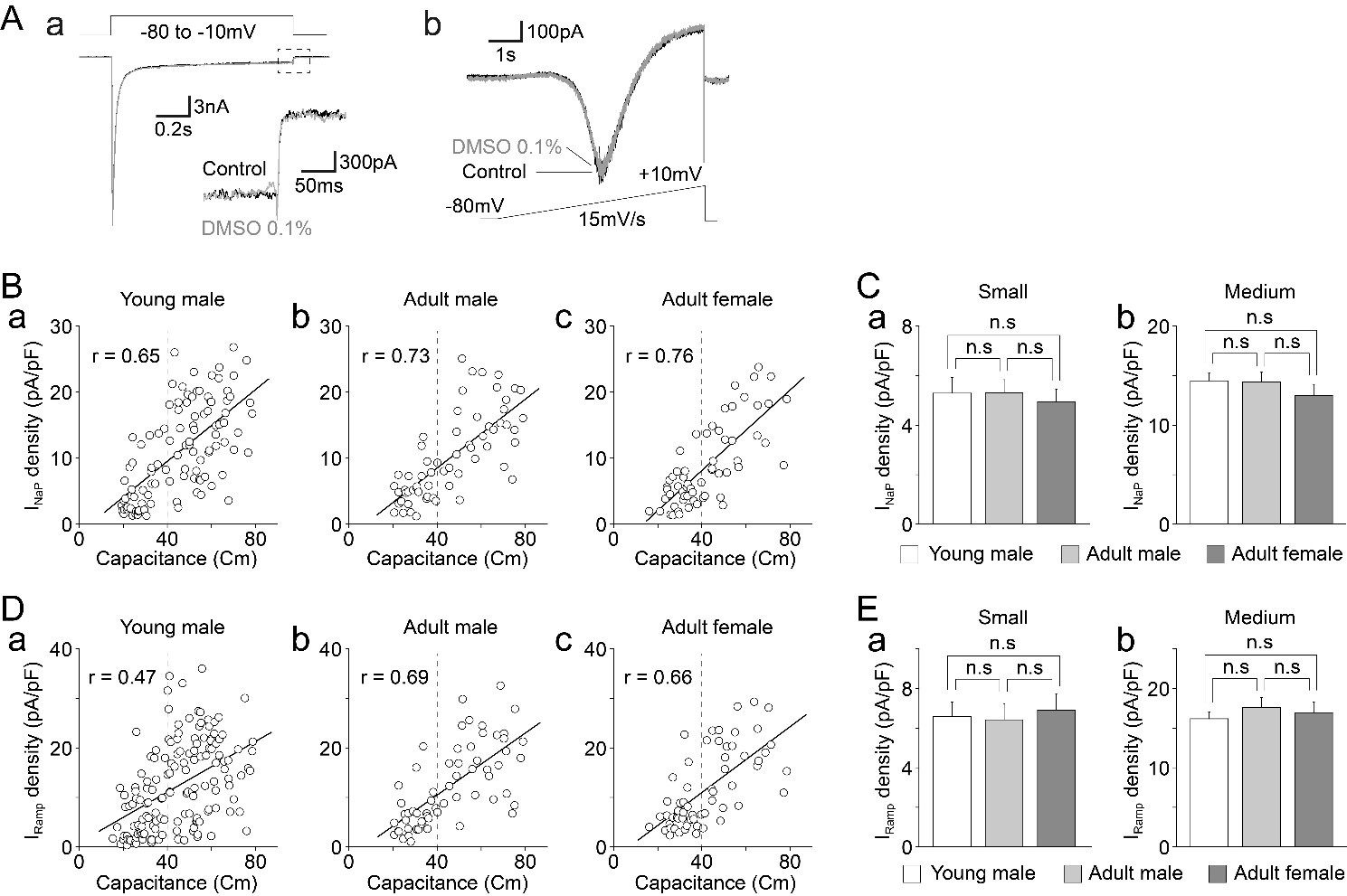


**Supplementary Fig. S1. Basal properties of TTX-R I_NaP_ and I_Ramp_ in adult male and female rats**

**A.** Typical traces of TTX-R I_NaP_ (**a**) and I_Ramp_ (**b**) in the absence and presence of 0.1% DMSO (v/v). Similar results were obtained from five independent experiments.

**B.** Scatter plots of the density of TTX-R I_NaP_ against membrane capacitance (Cm) obtained from DiI-positive neurons derived from young male (**a**, n = 71 neurons, same to Fig. 1D), adult male (**b**, n = 62 neurons), and adult female rats (**c**, n = 60 neurons). The linear trend lines represent the best fit using a least-squares fit (**a**; *r* = 0.65, **b**; *r* = 0.73, **c**; *r* = 0.76).

**C.** The mean values of the density of TTX-R I_NaP_ in small-sized (**a**; n = 31 neurons for young male, n = 28 neurons for adult male, and n = 31 neurons for adult female rats) and medium-sized (**b**; n = 40 neurons for young male, n = 34 neurons for adult male, and n = 29 neurons for adult female rats) DiI-positive neurons. The columns and error bars represent the mean and SEM. n.s; not significant (unpaired t-test).

**D.** Scatter plots of the density of TTX-R I_Ramp_ against membrane capacitance (Cm) obtained from DiI-positive neurons derived from young adult male (**a**, n = 152 neurons, same to Fig. 2Da), adult male (**b**, n = 62 neurons), and adult female rats (**c**, n = 60 neurons). The linear trend lines represent the best fit using a least-squares fit (**a**; *r* = 0.47, **b**; *r* = 0.69, **c**; *r* = 0.76).

**E.** The mean values of the density of TTX-R I_Ramp_ in small-sized (**a**; n = 60 neurons for young male, n = 28 neurons for adult male, and n = 31 neurons for adult female rats) and medium-sized (**b**; n = 92 neurons for young male, n = 34 neurons for adult male, and n = 29 neurons for adult female rats) DiI-positive neurons. The columns and error bars represent the mean and SEM. n.s; not significant (unpaired t-test).

**Figure S2**


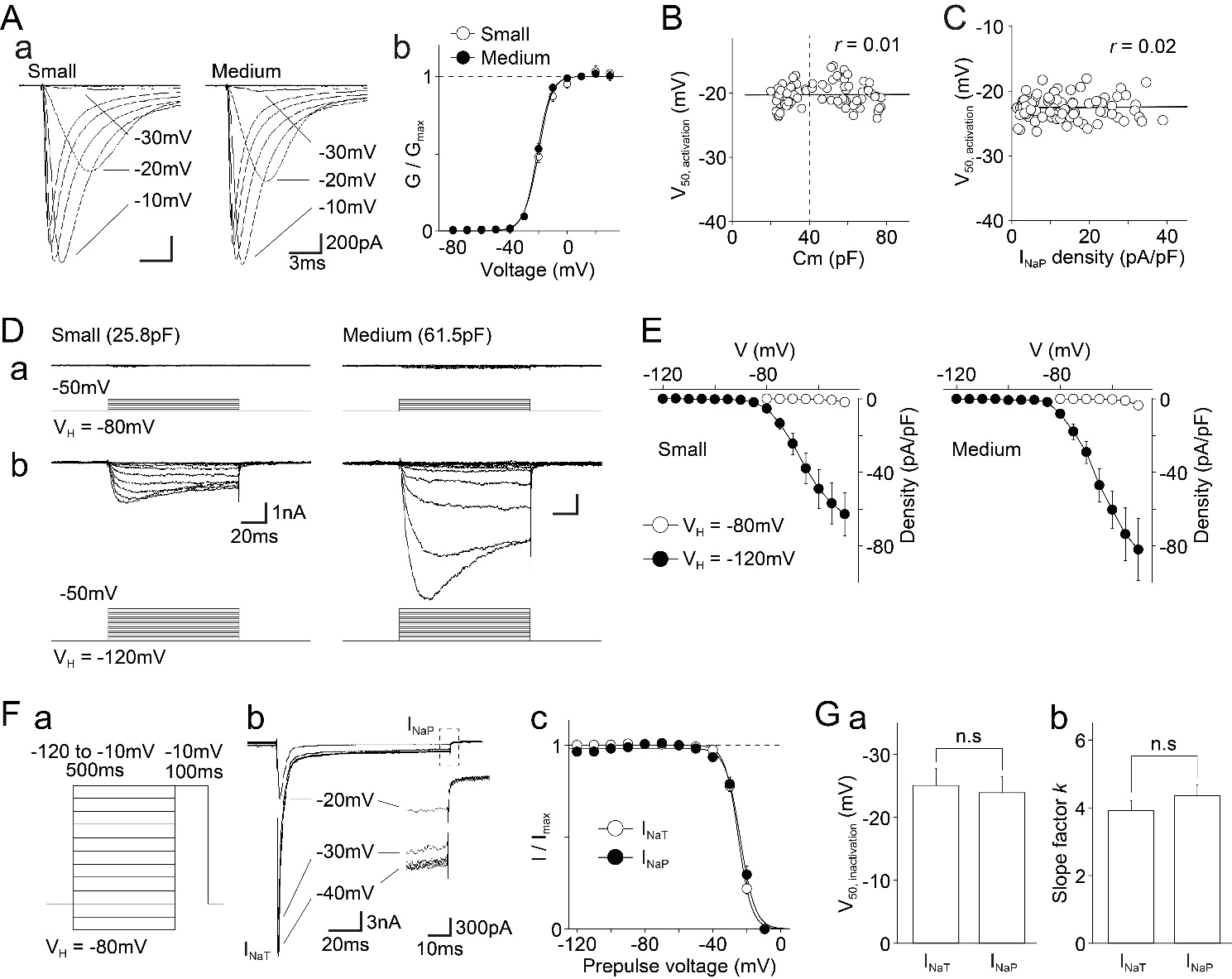


**Supplementary Fig. S2. Voltage dependence of TTX-R Na^+^ channels in dural afferent neurons**

**A.** **a**, Typical traces of TTX-R I_Na_ elicited by step pulses (100 ms depolarization pulses from -80 to +30 mV in 10 mV increments at a V_H_ of -80 mV) in small- (left) and medium-sized (right) DiI-positive neurons. **b**, Conductance-voltage relationships of TTX-R Na^+^ channels in small- (open circles) and medium-sized (closed circles) DiI-positive neurons. The points and error bars represent the mean and SEM from 32 small-sized and 44 medium-sized DiI-positive neurons. The continuous lines represent the best fits using a Boltzmann function.

**B.** Scatter plot of the half-maximal voltage for activation (V_50, activation_) against membrane capacitance (Cm) (n = 76). The linear trend line represents the best fit using a least-squares fit (*r* = 0.01).

**C.** Scatter plot of the half-maximal voltage for activation (V_50, activation_) against the density of TTX-R I_NaP_ (n = 76). The linear trend line represents the best fit using a least-squares fit (*r* = 0.02).

**D.** Typical traces of TTX-R I_Na_ elicited by step pulses (100 ms duration; up to -50 mV in 10 mV increments) at V_H_s of -80 mV (**a**) or -120 mV (**b**) in the same small- (left) and medium-sized (right) DiI-positive neurons. Notably, these slowly desensitizing I_Na_, which were mediated by Na_V_1.9, were elicited when neurons were held at a V_H_ of -120 mV but not of -80 mV.

**E.** Current-voltage relationships of TTX-R Na^+^ channels in small- (left) and medium-sized (right) DiI-positive neurons. The points and error bars represent the mean and SEM from eight small-sized and seven medium-sized DiI-positive neurons.

**F.** **a**, Schematic illustration of the voltage step stimulation for steady-state fast inactivation of TTX-R Na^+^ channels. **b**, Typical traces of TTX-R I_Na_ elicited by step pulses in medium-sized DiI-positive neurons. The inset represents the I_NaP_ region (dotted box) with an expanded time scale. **c**, Current-voltage relationships of TTX-R I_NaT_ (open circles) and I_NaP_ (closed circles) in medium-sized DiI-positive neurons. Each point represents the mean and SEM from 10 experiments. The continuous lines represent the best fits using a Boltzmann function.

**G.** Midpoint voltage for inactivation (V_50, inactivation_, **a**) and slope factor (**b**) of TTX-R I_NaT_ and I_NaP_. The columns and error bars represent the mean and SEM from 10 medium-sized DiI-positive neurons. n.s; not significant (paired t-test).

**Figure S3**


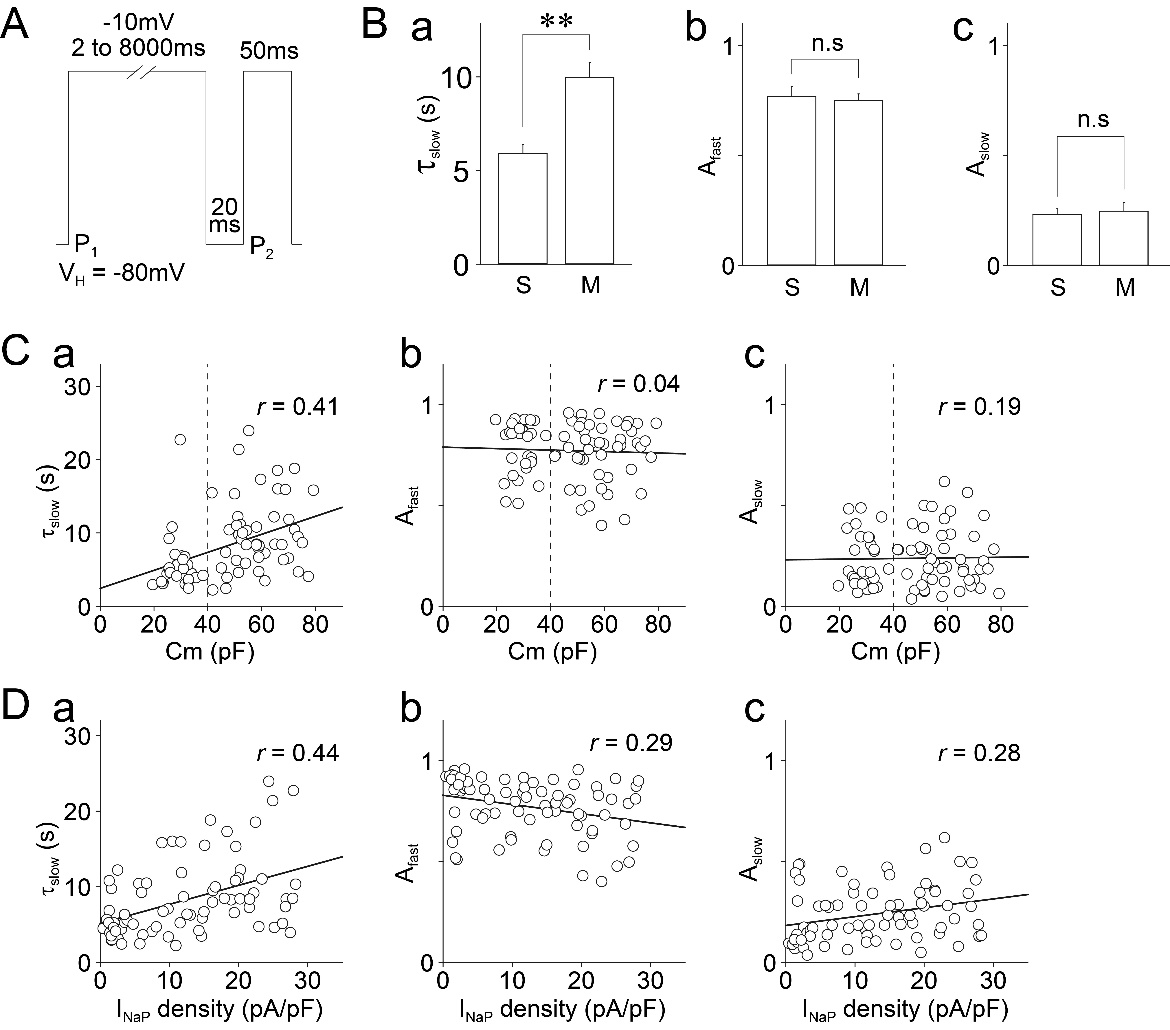


**Supplementary Fig. S3. Kinetic parameters for the development of inactivation of TTX-R Na^+^ channels in dural afferent neurons**

**A.** Schematic illustration of the two-pulse protocols used for the development of inactivation of TTX-R Na^+^ channels. TTX-R I_Na_ were induced by the conditioning prepulse (P_1_: -10 mV; 2–8,000 ms duration), which was followed by the test pulse (P_2_: -10 mV; 50 ms duration). The second TTX-R I_Na_ was recovered with an interpulse interval of 20 ms at a V_H_ of -80 mV.

**B.** The mean values of τ_slow_ (**a**), A_fast_ (**b**), and A_slow_ (**c**) in small- (S) and medium-sized (M) DiI-positive neurons. The columns and error bars represent the mean and SEM from 28 small- and 47 medium-sized DiI-positive neurons. **; p < 0.01, n.s; not significant (unpaired t-test).

**C.** Scatter plots of τ_slow_ (**a**), A_fast_ (**b**), and A_slow_ (**c**) against membrane capacitance (Cm) (n = 75). The linear lines represent the best fits using a least-squares fit.

**D.** Scatter plots of τ_slow_ (**a**), A_fast_ (**b**), and A_slow_ (**c**) against the density of TTX-R I_NaP_ (n = 75). The linear lines represent the best fits using a least-squares fit.

**Figure S4**


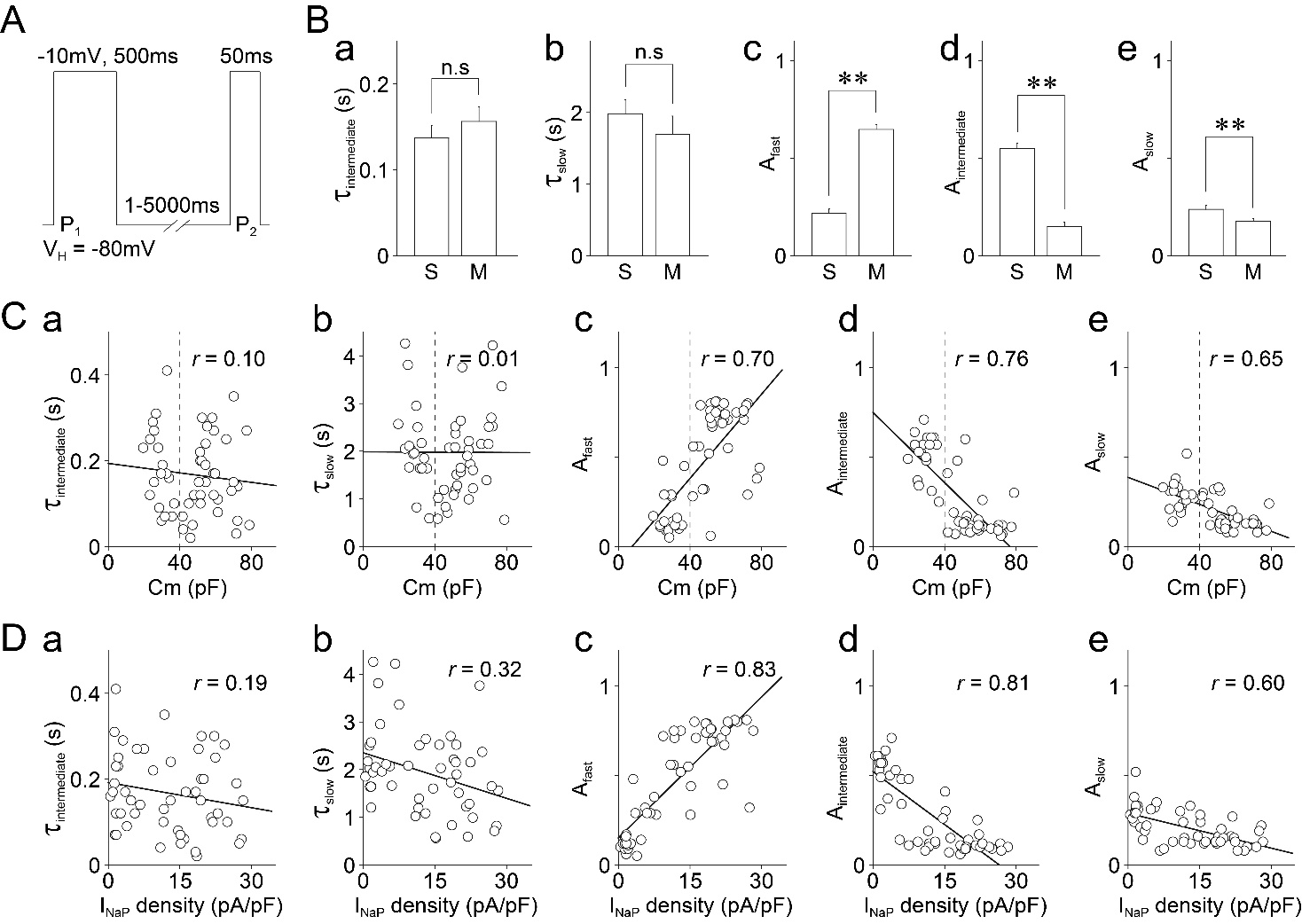


**Supplementary Fig. S4. Kinetic parameters for the recovery from inactivation of TTX-R Na^+^ channels in dural afferent neurons**

**A.** Schematic illustration of the two-pulse protocol used for the recovery from inactivation of TTX-R Na^+^ channels. TTX-R I_Na_ were induced by the conditioning prepulse (P_1_: -10 mV; 500 ms duration), which was followed by the test pulse (P_2_: -10 mV; 50 ms duration). The second TTX-R I_Na_ was recovered with various interpulse intervals of 1–5,000 ms at a V_H_ of -80 mV.

**B.** The mean values of τ_intermediate_ (**a**), τ_slow_ (**b**), A_fast_ (**c**), A_intermediate_ (**d**), and A_slow_ (**e**) in small- (S) and medium-sized (M) DiI-positive neurons. The columns and error bars represent the mean and SEM from 18 small-sized and 33 medium-sized DiI-positive neurons. **; p < 0.01, n.s; not significant (unpaired t-test).

**C.** Scatter plots of τ_intermediate_ (**a**), τ_slow_ (**b**), A_fast_ (**c**), A_intermediate_ (**d**), and A_slow_ (**e**) against membrane capacitance (Cm) (n = 51). The linear trend lines represent the best fits using a least-squares fit.

**D.** Scatter plots of τ_intermediate_ (**a**), τ_slow_ (**b**), A_fast_ (**c**), A_intermediate_ (**d**), and A_slow_ I against the density of TTX-R I_NaP_ (n = 51). The linear trend lines represent the best fits using a least-squares fit.

**Figure S5**


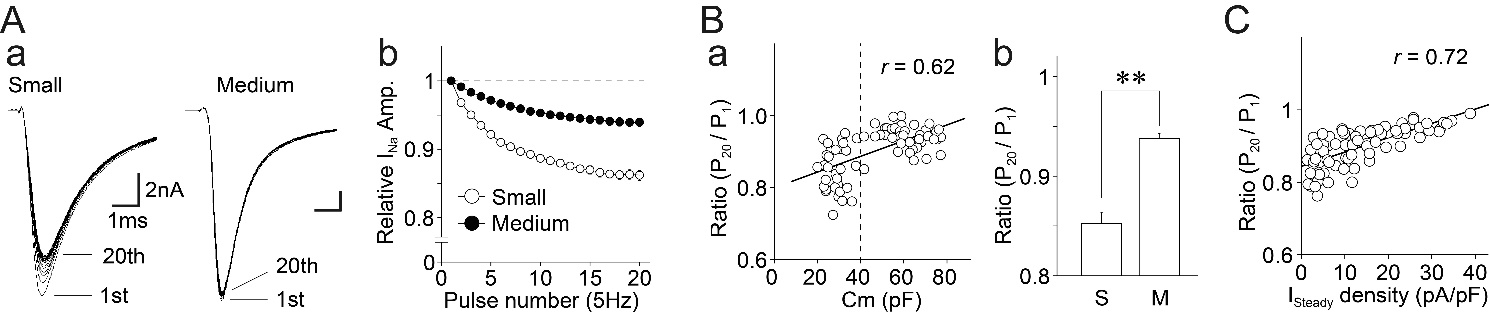


**Supplementary Fig. S5. Use-dependency of TTX-R Na^+^ channels in dural afferent neurons**

**A.** **a**, Typical traces of TTX-R I_Na_ elicited by 20 successive voltage step pulses (5 Hz; -10 mV; 30 ms duration) in small- (left) and medium-sized (right) DiI-positive neurons. **b**, Time course of the amplitude of TTX-R I_Na_ during a train of 20 pulses in small- (open circles) and medium-sized (closed circles) DiI-positive neurons. The peak amplitudes of TTX-R I_Na_ were normalized to the respective first amplitude and plotted against the pulse number. The points and error bars represent the mean and SEM from 32 small- and 44 medium-sized DiI-positive neurons.

**B.** **a**, Scatter plot of the P_20_/P_1_ ratio against membrane capacitance (Cm) (n = 76). The linear trend line represents the best fit using a least-squares fit (*r* = 0.62). **b**, The mean values of the P_20_/P_1_ ratio in small- (S) and medium-sized (M) DiI-positive neurons. The columns and error bars represent the mean and SEM from 32 small- and 44 medium-sized DiI-positive neurons. **; p < 0.01 (unpaired t-test).

**C.** Scatter plot of the P_20_/P_1_ ratio against the density of TTX-R I_NaP_ (n = 76). The linear trend line represents the best fit using a least-squares fit (*r* = 0.72).

**Figure S6**


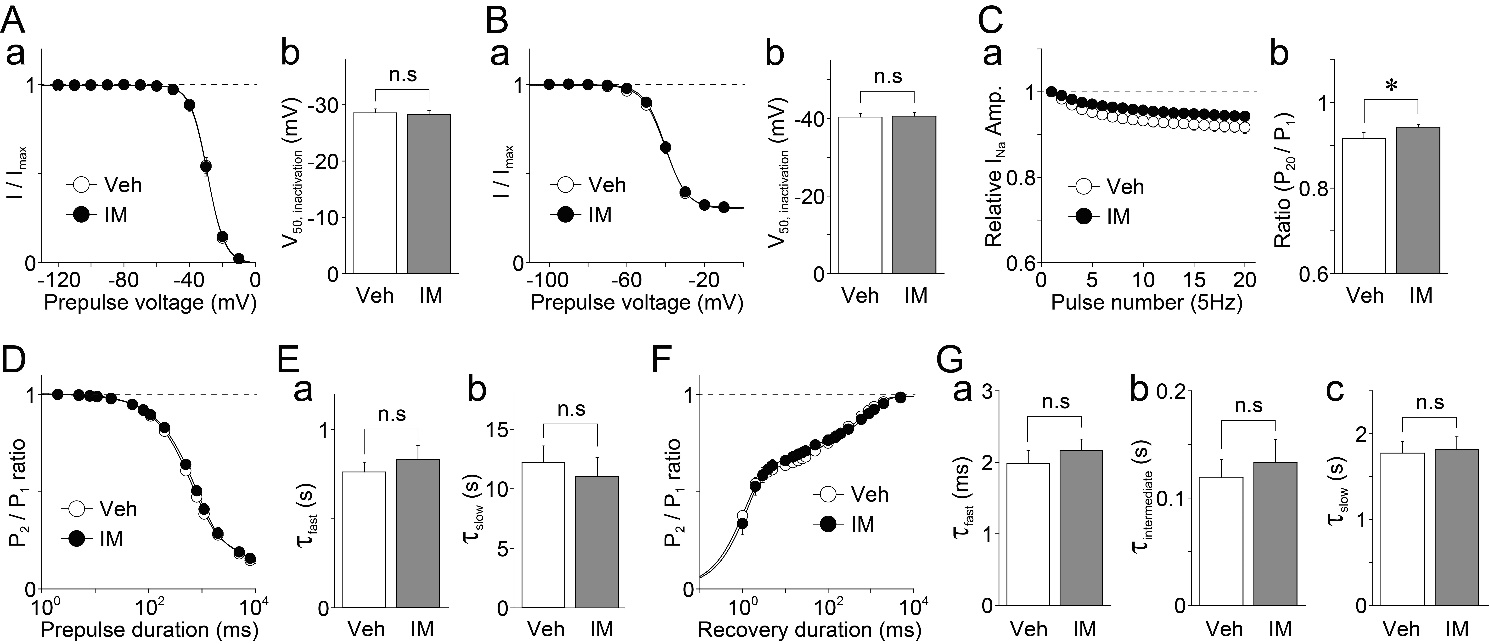


**Supplementary Fig. S6. Inflammatory mediator-induced changes in the properties of TTX-R Na^+^ channels in medium-sized dural afferent neurons**

**A.** **a**, Voltage-fast inactivation relationships of TTX-R Na^+^ channels in vehicle-treated (open circles) and IM-treated (closed circles) medium-sized DiI-positive neurons. Each point represents the mean and SEM from 25 vehicle-treated and 18 IM-treated medium-sized DiI-positive neurons. The continuous lines represent the best fits using a Boltzmann function. **b**, The mean values of the V_50, inactivation_ in vehicle- and IM-treated medium-sized DiI-positive neurons. The columns and error bars represent the mean and SEM from 25 vehicle-treated and 18 IM-treated medium-sized DiI-positive neurons. n.s; not significant (unpaired t-test).

**B.** **a**, Voltage-slow inactivation relationships of TTX-R Na^+^ channels in vehicle-treated (open circles) and IM-treated (closed circles) medium-sized DiI-positive neurons. The points and error bars represent the mean and SEM from 21 vehicle-treated and 18 IM-treated medium-sized DiI-positive neurons. The continuous lines represent the best fits using a Boltzmann function. **b**, The mean values of V_50, inactivation_ in vehicle- and IM-treated medium-sized DiI-positive neurons. The bars and errors represent the mean and SEM from 21 vehicle-treated and 18 IM-treated medium-sized DiI-positive neurons. n.s; not significant (unpaired t-test).

**C.** **a**, Time course of the amplitude of TTX-R I_Na_ during a train of 20 pulses in vehicle-treated (open circles) and IM-treated (closed circles) medium-sized DiI-positive neurons. The peak amplitudes of TTX-R I_Na_ were normalized to the respective first amplitude and plotted against the pulse number. The points and error bars represent the mean and SEM from 23 vehicle-treated and 14 IM-treated medium-sized DiI-positive neurons. **b**, The mean values of the P_20_/P_1_ ratio for vehicle- and IM-treated medium-sized DiI-positive neurons. The columns and error bars represent the mean and SEM from 23 vehicle-treated and 14 IM-treated medium-sized DiI-positive neurons. *; p < 0.05 (unpaired t-test).

**D.** Kinetics for the development of inactivation of TTX-R Na^+^ channels in vehicle-treated (open circles) and IM-treated (closed circles) medium-sized DiI-positive neurons. The P_2_/P_1_ ratio of TTX-R I_Na_ was plotted against the duration of the conditioning prepulse. The points and error bars represent the mean and SEM from 25 vehicle-treated and 18 IM-treated medium-sized DiI-positive neurons. The continuous lines represent the best fits using a double exponential function.

**E.** The mean values of τ_fast_ (**a**) and τ_slow_ (**b**) in vehicle- and IM-treated medium-sized DiI-positive neurons. The columns and error bars represent the mean and SEM from 25 vehicle-treated and 18 IM-treated medium-sized DiI-positive neurons. n.s; not significant (unpaired t-test).

**F.** Kinetics of the recovery from inactivation of TTX-R Na^+^ channels in vehicle-treated (open circles) and IM-treated (closed circles) medium-sized DiI-positive neurons. The P_2_/P_1_ ratio of TTX-R I_Na_ was plotted against the recovery duration. The points and error bars represent the mean and SEM from 25 vehicle-treated and 18 IM-treated medium-sized DiI-positive neurons. The continuous lines represent the best fits using a triple exponential function.

**G.** The mean τ_fast_ (**a**), τ_intermediate_ (**b**), and τ_slow_ (**c**) values in vehicle- and IM-treated medium-sized DiI-positive neurons. The columns and error bars represent the mean and SEM from 25 vehicle-treated and 18 IM-treated medium-sized DiI-positive neurons. n.s; not significant (unpaired t-test).

**Table S1**

**Supplementary Table S1. Basal membrane properties of C-type dural afferent neurons.**

|  | **C_m_ (pF)** | **RMP (mV)** | **R_in_ (MΩ)** | **Rheobase (pA)** | **Firing pattern** ^†^ **(%)** | | | **n** |
| --- | --- | --- | --- | --- | --- | --- | --- | --- |
|  |  |  |  |  | **Single** | **Phasic** | **Tonic** |  |
| **Small** | 30.8 ± 1.0 | -53.5 ± 1.9 | 482.9 ± 64.7 | 144.8 ± 11.4 | 4 (16.0) | 18 (72.0) | 3 (12.0) | 25 |
| **Medium** | 58.9 ± 2.1 | -51.8 ± 1.2 | 308.6 ± 36.1 | 103.9 ± 8.3 | 4 (12.9) | 0 (0.0) | 27 (87.1) | 31 |

†; Firing patterns were determined by voltage responses elicited by 4-fold threshold currents.

**Table S2**

**Supplementary Table S2. Basal membrane properties of vehicle-treated and IM-treated C-type dural afferent neurons.**

|  |  | **C_m_ (pF)** | **RMP (mV)** | **R_in_ (MΩ)** | **Rheobase (pA)** | **Firing pattern** ^†^ **(%)** | | | **n** |
| --- | --- | --- | --- | --- | --- | --- | --- | --- | --- |
|  |  |  |  |  |  | **Single** | **Phasic** | **Tonic** |  |
| **Small** | **Vehicle** | 30.3 ± 1.4 | -53.6 ± 7.3 | 440.9 ± 71.2 | 146.7 ± 23.8 | 1 (8.3) | 9 (75.0) | 2 (16.7) | 12 |
|  | **IM** | 28.7 ± 1.5 | -52.5 ± 2.1 | 506.8 ± 47.1 | 96.9 ± 13.2^*^ | 1 (7.7) | 9 (69.2) | 3 (23.1) | 13 |
| **Medium** | **Vehicle** | 57.1 ± 2.6 | -51.5 ± 7.8 | 243.4 ± 55.9 | 92.0 ± 15.6 | 1 (10.0) | 0 (0.0) | 9 (90.0) | 10 |
|  | **IM** | 55.6 ± 2.5 | -51.8 ± 1.8 | 265.0 ± 35.9 | 87.5 ± 9.8 | 1 (8.3) | 0 (0.0) | 11 (91.7) | 12 |

†; Firing patterns were determined by voltage responses elicited by 4-fold threshold currents.

*; p < 0.05.
